# Translational Preclinical Pharmacologic Disease Models for Ophthalmic Drug Development

**DOI:** 10.1007/s11095-019-2588-5

**Published:** 2019-02-25

**Authors:** Mihir Shah, Sara Cabrera-Ghayouri, Lori-Ann Christie, Katherine S. Held, Veena Viswanath

**Affiliations:** Biological Research, Allergan plc, 2525 Dupont Drive, Irvine, California 92612 USA

**Keywords:** Age-related macular degeneration, diabetic retinopathy, dry eye disease, glaucoma, ocular allergy

## Abstract

Preclinical models of human diseases are critical to our understanding of disease etiology, pathology, and progression and enable the development of effective treatments. An ideal model of human disease should capture anatomical features and pathophysiological mechanisms, mimic the progression pattern, and should be amenable to evaluating translational endpoints and treatment approaches. Preclinical animal models have been developed for a variety of human ophthalmological diseases to mirror disease mechanisms, location of the affected region in the eye and severity. These models offer clues to aid in our fundamental understanding of disease pathogenesis and enable progression of new therapies to clinical development by providing an opportunity to gain proof of concept (POC). Here, we review preclinical animal models associated with development of new therapies for diseases of the ocular surface, glaucoma, presbyopia, and retinal diseases, including diabetic retinopathy and age-related macular degeneration (AMD). We have focused on summarizing the models critical to new drug development and described the translational features of the models that contributed to our understanding of disease pathogenesis and establishment of preclinical POC.

## INTRODUCTION

The pathologies of the eye are usually divided into different categories based on the affected region: ocular surface, anterior and posterior diseases. Multiple diseases such as cataract, age-related macular degeneration (AMD), glaucoma, presbyopia, dry eye disease (DED), ocular allergy and others can lead to visual impairments and blindness. Ocular pathologies are also observed as a common finding in multiple systemic diseases including autoimmune diseases like rheumatoid arthritis, Sjogren’s Syndrome (SS), rosacea, Graves Diseases, graft *versus* host disease and chronic diseases such as diabetes and hypertension. Development of therapeutic strategies for ocular diseases requires a thorough understanding of etiology, molecular and anatomical pathogenic mechanisms and natural history of the disease. Mimicking human diseases in animal models in species such as mice, rats, guinea pigs, rabbits, dogs, and primates has been critical to our understanding of several human ocular diseases and for the development of novel drugs, drug delivery strategies and advancement of ophthalmological diagnostic technologies. This review provides a focused summary of models key to the development of novel therapies for the most common ocular diseases (ocular allergy, DED, glaucoma, presbyopia, diabetic retinopathy (DR), and AMD). The application of these models in ocular pharmacology and translational potential to human disease are also discussed. Our intention is not to provide a complete listing of all preclinical models for these ophthalmic diseases. Instead, we focus on the key *in vivo* models that were used to establish preclinical POC prior to entry into the clinic, with emphasis on drugs that have demonstrated clinical efficacy. Collation of drugs and their preclinical POC models was done by reviewing information provided in regulatory documents (eg. New Drug Applications, NDA), conference abstracts, patent publications, company press releases, Clarivate Analytics’ Integrity Experimental Models Knowledge Area and published literature. Although many of these animal models mimic aspects of ocular diseases, none of them capture the full complexity of human disease. Therefore, multiple models of the same disease are typically needed to recapitulate different aspects of human pathology. Finally, we outline several gaps in current preclinical models and endpoints that should be considered in the future development of novel therapeutics to address unmet patient needs.

### Ocular Surface Diseases

The ocular surface is a complex tissue system harmonized to achieve a stable tear film and contribute to visual acuity. The lacrimal and meibomian glands, mucin producing goblet cells, and nervous systems are key components of a lacrimal functional unit which contribute to maintaining a stable tear film (1). Furthermore, epithelial cells and underlying lymphoid associated tissues provide a mucosal barrier to prevent irritants and pathogens from entering the body. The ocular surface is vulnerable to environmental insults and when components of this tissue are compromised mild to severe disease can ensue. The most prevalent ocular surface disorders encompass dry eye disease (DED), blepharitis, meibomian gland dysfunction (MGD), and *ocular* allergy. Patients may experience ocular dryness, itch, photosensitivity and foreign body sensations, which in many cases are shared symptoms of several ocular surface disorders (2). Disease progression necessitates pharmaceutical intervention such as anti-inflammatory drugs for DED and ocular allergy, and antibiotics for blepharitis. While many patients respond to pharmaceutical treatments, there remains a large population that is non-responsive or cannot tolerate the side effects associated with these therapies. To enable effective therapies a variety of *in vivo* models that mimic multi-tissue heterogeneous pathologies have been developed. Highlighted in this portion of the review are animal models of ocular allergy, dry eye syndrome (aqueous deficient and evaporative), and ocular pain that have been utilized in drug development. We will not address preclinical models of ocular infections or wound healing in the eye as they have been reviewed elsewhere (3,4).

#### Ocular Inflammation: Ocular Allergy and Dry Eye Disease

Inflammation plays a key role in the pathology of multiple diseases of the ocular surface including DED, SS and ocular allergy, thus desirable drug candidates include anti-inflammatory or immune modulatory molecules. A variety of preclinical ocular models have been used to validate the anti-inflammatory property of drugs, as one model may not represent all aspects of the pathology. Discussed below are the preclinical models used in drug development for ocular allergy and DED. Multiple models of SS have been reported in the literature which have been previously reviewed and will not be addressed in the present review (5,6).

##### Ocular Allergy

Ocular allergy (allergic conjunctivitis) is a hypersensitivity disorder that affects the conjunctiva, lids and/or cornea, with prevalence as high as 20% in the U.S. (7). Several subtypes have been described including: Seasonal, the prominent subtype that results in mild disease, perennial allergic conjunctivitis, and the less common severe vernal and atopic keratoconjunctivitis disorders (VKC and AKC). Robust clinical signs and symptoms such as tearing, chemosis, mucous discharge and itch are shared among the subtypes, while severe allergy may involve keratitis and corneal ulcer (8). Ocular allergic reactions are initiated with IgE-mediated responses that result in mast cell degranulation of factors that drive the hallmark signs and symptoms. This early-phase response peaks within 1 h, and can lead to a late-phase response involving the influx of eosinophils and neutrophils 4–6 h later (9). While mild ocular allergy is usually self-resolving and does not compromise vision, the elevated T cell and eosinophil mediated immunopathology in VKC and AKC is associated with chronic disease that can lead to vision impairment. Therapy involves identification and avoidance of allergen, while, pharmaceutical treatment options mainly include antihistamine and mast cell stabilizer topical/systemic drugs, and topical steroids may be used in severe disease states. Next generation anti-inflammatory options aim to lengthen the drug effect of antihistamines, and limit drug side effects of standard of care anti-inflammatories such as steroids, which are known to induce glaucoma.

##### Allergy Therapies

Several preclinical models in rodents, rabbit and dog have been described which capture aspects of allergic response phase and ocular allergy disease severity (10). Primary pharmacodynamic activity for fast acting anti-inflammatory drugs which target histamine H1 receptor and/or mast cell stabilization or alternative immune modulatory mechanisms have been supported using histamine treatment, compound 48/80 treatment, and antigen-challenge induction models (11,12). Anaphylactic reaction mediated by passive anti-ovalbumin treatment produces increased vascular permeability and clinically translatable signs of chemosis, edema and discharge. Likewise, histamine topical ophthalmic treatment induces vascular permeability within the conjunctiva. These methods of ocular allergic reaction are rapid and transient, yet have aided in early drug development, such as with AL-4943A (olopadatine, Alcon) drug dosing studies in rats and guinea pigs (12). Rapid induction of ocular allergy can also be done using compound 48/80, a non-immunogenic mast cell degranulatory agent, that produces chemosis, tearing, mucous discharge, and itch-responses in animals and humans (13). Topical treatment with compound 48/80 leads to a robust granulocyte infiltration, rapid histamine response that peaks within 1-h post-treatment, with sustained inflammation mediated by T cells for up to three days in humans and animals (13,14). Antihistamine activity of Lastacaft® (alacaftadine, Allergan) was successfully demonstrated in this model using New Zealand white rabbits (11). In addition, 48/80-induced allergic reaction has translated to the clinic and was used to assess efficacy of a second-generation histamine H1 receptor antagonist levocabastine administered therapeutically to patients (15).

In contrast to models that mainly capture mast cell degranulatory responses, antigen-challenge models provide a better opportunity to evaluate factors associated with chronic disease. Induction of antigen-challenge begins with a sensitization period initiated with peripheral immunization, typically with a protein, ovalbumin, or an environmental antigen, ragweed. This is followed by topical antigen challenge that induces chemosis, tearing, mucus discharge, redness, and itch-response (16). Similar to the cellular players observed with compound 48/80 induction, antigen-challenge stimulation paradigm results in a more pronounced conjunctival eosinophil infiltration and T helper type 2 (Th2) cell mediated immunopathology is maintained (17). In addition, other cell types have been described including the interplay of polymorphonuclear cells and T helper type 17 cells (Th17) cells (18,19). Recently, a severe model called allergic eye disease (AED) was developed in mice which produces sustained clinical signs of conjunctival fibrosis, cornea lymphangiogensis, and higher IgE levels than traditional models of allergic conjunctivitis. (18,20–22). The AED model was implemented in the development of Isunakinra (EBI-005, Eleven Biotherapeutics), an IL-1R antagonist. IL-1 is a master regulator of ocular inflammation and plays a role in the pathogenesis of both acute and chronic disease. Isunakinra showed efficacy when delivered topically in AED and another ocular surface disease model of DED. However, Phase 3 clinical endpoints were not met for Isunakinra highlighting translational challenges (23,24). It will be telling if a non-traditional novel anti-inflammatory treatment will be successful in the clinic, and support translatability of these antigen-challenge models. Additional approaches could involve sensitization and challenge via topical routes to better mimic clinical conjunctival antigen challenge models, and importantly, models which involve keratitis or cornea ulceration to reproduce signs observed in more severe forms of chronic human ocular allergy (16,25).

##### Dry Eye Disease

Dry eye is a multifactorial disease of the ocular surface which can result in a spectrum of symptoms and/or signs that affects 5–30% of the population (26). Commonly dry eye results in symptoms of discomfort and visual disturbances that are described as eye dryness, foreign body sensation, grittiness, light sensitivity, and pain. The common signs of DED include diminished tear volume, increased ocular surface staining, reduce tear break-up time, and abnormal meibomian glands. Central to DED is tear hyperosmolarity that can initiate inflammation and lead to ocular surface damage, which creates a vicious cycle (27). Classically, dry eye is organized into tear-deficient and evaporative forms that relate to insufficient tear production, and loss of tear stability and evaporation, respectively. DEWS report defines DED to include inflammation and neurosensory abnormalities as etiologic roles (28,29). Another factor contributing to the loss of tear film homeostasis is MGD, which is the most frequent cause of DED (30).

Dry eye treatment strategies include artificial tear substitutes, gels/ointments, moisture chamber spectacles, topical anti-inflammatory agents, tetracyclines, punctual plugs, tear and mucin secretagogues, serum, systemic immunosuppressants, and surgical alternatives (31,32). Ocular pain and discomfort are also a major part of DED and there are therapeutic strategies being developed to address this aspect of the disease. Several preclinical models have been instrumental in early development of DED therapies. Organized below are sections reviewing preclinical models associated with the development of topical anti-inflammatory, secretagogue, and ocular pain therapies.

##### Anti-Inflammatory Therapies

Inflammation contributes to the pathogenesis of DED and chronicity of symptoms, regardless of the etiological factors underlying the disease. Inflammation causes an array of changes to the ocular surface that are similar in humans and animal models: loss of corneal epithelial integrity, decrease in goblet cell density, lacrimal gland damage, altered tear production and composition, and aberrant corneal nerves (30). Several animal models have been used to lay the foundation to elucidate immunopathology observed in tear deficient and evaporative forms of DED, and detailed descriptions of these models have been previously reviewed (33,34). Although many animal models of dry eye have been generated, only a handful have been translational in anti-inflammatory drug development and will be reviewed below.

Currently, there are two approved anti-inflammatory drugs for DED; RESTASIS® (cyclosporine, Allergan), and XIIDRA® (lifitegrast, Shire). Lifitegrast suppresses inflammation by inhibiting integrin interaction between ICAM-1: LFA-1. This interaction functions in cell migration and formation of the immunological synapse that facilitate T cell activation (35). Anti-inflammatory efficacy was observed in a non-dry eye preclinical evaluation of lifitegrast using the rat streptozotocin (STZ) model of DR to evaluate retinal leukostasis and blood-retinal barrier breakdown (36). Following approval, the efficacy of lifitegrast was observed in a mouse desiccating stress model where aqueous tear deficiency was induced by administering scopolamine, and tear evaporation was induced by exposure to low humidity plus air flow environmental conditions (37). Dursun *et al*. have characterized this model and observed alterations to aqueous tear production, corneal barrier function, conjunctiva morphology and goblet cell density all of which are also features of human DED (38). The lifitegrast-treated group had significantly lower expression of T helper type 1 cytokines family genes, less corneal barrier disruption, and greater conjunctival goblet cell density/area, compared to vehicle-treated mice. Furthermore, the desiccating stress model was used in preclinical efficacy studies of other anti-inflammatory drugs such as tofacitinib (CP-690,550, Pfizer) a JAK/STAT pathway inhibitor, and the IL-1R antagonist, EBI-005 mentioned above. Topical treatment with CP-690,550 or EBI-005, in mice exposed to desiccating stress resulted in a significant decrease in corneal staining and/ or expression of inflammatory mediators in cornea and conjunctival tissues, relative to vehicle (24,39). A similar evaluation of inflammatory mediators was conducted in the clinic using DED patients tears in the clinical study that evaluated CP-690,550 (40).

Variations to the desiccating stress model have been explored which reveal unique immunopathology and ability to mimic chronic disease. Research by Chen *et al*. showed that different effector T cell populations contributed to disease when comparing pharmacological and environmental stress induction methods (41). Chen *et al*. later explored the chronic phase of the desiccating stress model and showed that elevated corneal fluorescein staining scores were maintained for 16 weeks post-desiccating stress. Furthermore, evaluation of the immunopathology revealed memory Th17 cells as critical inducers of the chronic and relapsing course of DED, which was demonstrated with adaptive transfer into recipients prior to environmental stress-only exposure (42). While the desiccating model of DED continues to provide valuable understanding of immunopathology of DED and advancements have been made in chronic paradigms that better mimic DED in human, evaluation of drug efficacy in higher species, with larger eyes, can provide a better mode to assess pharmacokinetic-pharmacodynamic (PK-PD) relationships.

The key model reported in the lifitegrast NDA is the dog model of dry eye: canine keratoconjunctivitis sicca (KCS) (43). Canine KCS is a spontaneous disease with approximately 1% prevalence and shares similar clinical signs with human DED such as reduced tear production, eye irritation, epitheliopathy, as well as similar mechanisms of immunopathology (44). In fact, topical ophthalmic delivery of cyclosporine to dogs with KCS has already been successfully implemented in veterinary ophthalmology, creating a path for early development of Restasis® and its final approval in 2002. Topical cyclosporine treatment of canine KCS resulted in ameliorated keratitis and conjunctivitis, with modulated cytokine expression and diminished cellular infiltration to the lacrimal gland (45,46). These studies highlight key features of KCS immunopathology in dogs that have also been described in human DED: Increased T cell infiltration in the lacrimal gland and conjunctiva, increased apoptosis of lacrimal gland and conjunctiva epithelial cells, and suppressed apoptosis of T cells within these tissues (47–52). Lifitegrast was evaluated in KCS dogs in a 12-weeks treatment study. Treatment resulted in a significant increase in tear production from baseline, which corresponded with decreased inflammatory cell infiltrate of the conjunctiva relative to baseline (53). While these findings indicate dog KCS model is a validated approach to test broad-acting anti-inflammatory drugs for DED, the immunopathogenesis of canine KCS is not completely defined and the signs of disease tend to be more severe in dogs than what is observed in DED patients. Dog KCS can have pronounced conjunctival hyperemia, thick mucoid discharge, recurrent ulcers, and systemic effects similar to those observed in human SS patients (54). Furthermore, understanding of immunopathology can be improved by a thorough evaluation of tear cytokine expression, which may provide a translational readout in the clinic. Finally, there are challenges to this model such as acquiring a colony of dogs with a spontaneous disease, and enrolling subjects for immunopathology and/or drug studies as they are often pets, thus considerations of study-commitment and sampling options will limit the use of dogs in early stages of drug discovery.

Beyond models of DED, drug development opportunities for anti-inflammatory targets can be achieved with other ocular inflammation models such as experimental uveitis. Experimental autoimmune anterior uveitis (EAAU) and endotoxin-induced uveitis (EIU) can serve as surrogate models for DED as anterior inflammation mechanisms are similar to those in dry eye and the disease can be modulated via topical delivery of drugs. EAAU can be induced in rats with immunization to melanin-associated antigen that stimulates a robust and reproducible macrophage and T cell-mediated uveitis approximately 2 weeks post-immunization and is sustained for an additional week. Clinical examination of uveitis and evaluation of aqueous humor cellular infiltrates, protein exudation, and pro-inflammatory cytokine and chemokine content can be explored and compared to anti-inflammatory positive control treatments such as cyclosporine and steroids (55). While EAAU provides an option for treatment duration up to 3 weeks, EIU model, in contrast, is self-resolving acute inflammation model that can be assessed in 48–72 h. EIU can be induced in rodents by LPS, endotoxin, injection to the intravenous, intraperitoneal, intravitreal or footpad routes and evokes an acute inflammation of the anterior chamber that peaks at approximately 24 h. post-injection. Similar to EAAU, aqueous humor inflammation can be examined, however, EIU cellular responses are mediated mainly by polymorphonuclear cells and macrophages of the innate immune system (56). The EIU model has successfully been used to demonstrate anti-inflammatory properties of a clinical stage narrow spectrum kinase inhibitor, TOP1362 (Topivert), compared to a topical steroid (57). TOP1362 and the steroid dose-dependently reduced LPS-induced inflammatory cell infiltration and ocular pro-inflammatory cytokine expression. Given the complexity of drug development for DED and the limitations of the available preclinical tools, it is evident that multiple models are needed to recapitulate the human disease and to generate preclinical POC to increase probability of success for the molecule in the clinic.

##### Secretagogues

It is well accepted that DED involves a complex mechanism that involves hyperosmolarity, tear instability and insufficiency, mucin loss in addition to the inflammatory mechanisms discussed above. Tears play a critical role in the etiology, progression and pathogenesis of DED and a stable tear film consists of a mucin layer, an aqueous layer and a lipid layer where the lacrimal and the Meibomian glands play a critical role (58). Treatments for tear insufficiency include artificial tears or lubricant agents, biological tear substitutes, “secretagogues” or in some cases use of punctal plugs to delay tear clearance.

In addition to the lacrimal gland, recently it has also been demonstrated that the ocular surface epithelia might be key modulators of basal tear volume and composition via cAMP-and calcium-dependent Cl- transporters, and by sodium (Na+) absorption through the epithelial Na + channel (ENaC) (59). Based on these observations, a novel approach to improve hydration on the ocular surface through the inhibition of sodium absorption with epithelial sodium channel (ENac) inhibitors developed by Parion Sciences has recently been shown to be effective in early stage clinical studies and is currently undergoing Phase 2/3 clinical studies (60). The progress of molecules in this mechanism was based on key preclinical studies that showed that ENaC blockers can provide durable increases in ocular surface hydration providing a novel mechanism for treatment of dry eye. The lead compound was tested topically in normal mice to evaluate effects on tear output and was shown to increase tears with a duration longer than observed with purinergic agonists. An acute model of aqueous deficient dry eye was utilized where dysfunction of the lacrimal gland was induced by injection of the pro-inflammatory cytokine interleukin 1 into the exorbital lacrimal gland which has been shown to induce severe inflammatory response, stimulate acinar and ductal cell proliferation, increase corneal fluorescein staining and inhibit protein secretion as well as tear production (61). It was demonstrated that ENaC inhibitors showed enhanced tear output and lower corneal fluorescein staining scores in this model.

An important class of drugs that have been developed that are aimed at improving tear homeostasis are the class of drugs known as secretagogues. Among the tear-mucin secretagogues two were evaluated in multiple preclinical models of aqueous deficient dry eye and have been launched in Japan. These include Mucosta® (rebamipide, Otsuka Pharmaceuticals) a novel quinolone derivative and Diquas® (diquafasol, Santen) a purinergic agonist (62). Rebamipide, whose detailed mechanism of action is not fully understood, has been shown to have mucosal protective properties, promote re-epithelialization of damaged tissue, and plays a role in inflammation resolution. A 2% ophthalmic suspension of this drug was approved in 2012 based on preclinical studies where it showed an effect on mucins, goblet cells and ocular surface inflammation. Diquafosol is a uridine dinucleotide analog which acts as purinergic P2Y2 receptor agonist and a 3% ophthalmic solution is approved as a therapy for dry eye in Japan. Activation of the P2Y2 receptors at the ocular surface increases tears and mucin production and has demonstrated benefits in patients with tear film instability (63). Both secretagogues have been evaluated in the superoxide dismutase-1 (SOD-1) knockout mice which shows decreased tear secretion due to oxidative and inflammatory stress to the lacrimal gland resulting atrophy and fibrosis that progresses with age (64–67). Rebamipide and diquafosol were evaluated in 40-week-old male SOD−/− mice and were both shown to improve tear stability, corneal epithelial damage, conjunctival goblet cell density and MuC5 messenger RNA expression. This model therefore represents an interesting approach to test therapeutic strategies that promote anti-oxidant defense systems and reduce oxidative stress-induced damage.

Several different variations of the mouse model in which lacrimal function is disrupted have been reported. In rodents, there are two distinct anatomical divisions of the lacrimal glands: the extraorbital gland on the temporal side of the eye, and the smaller intraorbital gland located beneath the bulbar conjunctiva of the outer canthus. Stevenson W *et al*, 2014 performed surgical excision of the extraorbital lacrimal gland in mice to induce a reproducible and lasting effect on aqueous tear secretion, severe corneal epitheliopathy and ocular surface inflammation and immunity (68). It has also been demonstrated that the mucosal tolerance can be disrupted by addition of ovalbumin, a well-known inert antigen, on the ocular surface in this model (69). To mimic a more severe phenotype of aqueous deficiency another model of lacrimal insufficiency has recently been reported in mice which involves excision of both exoribital and intraorbital lacrimal glands resulting in a severe, long lasting (over 10–12 weeks post-surgery) phenotype (70). This model has not yet been utilized to show reversal of disease with a therapeutic agent which will be critical to demonstrate its utility in translational predictability of the model. Lacrimectomy has also been performed in rats to generate a model of tear-deficient dry eye (71–73). Diquafosol and ENaC inhibitor described above have been evaluated in this model of lacrimal excision induced aqueous-deficient dry eye disease in rats and were shown to improve tear secretion and restore corneal epithelial barrier function (71,74). It has also been reported that reduction in tear volume observed in this model was not significantly improved by treatment with Restasis or dexamethasone (72). In contrast, the corneal surface damage and inflammatory mediators observed was attenuated by treatment with these anti-inflammatory drugs. Hence this model of lacrimal excision might have utility in differentiating between therapeutic mechanisms that are anti-inflammatory *versus* secretagogues.

Rebamipide and diquafosol were also tested in normal and ocular disease rabbits. In normal rabbits the drugs applied topically were shown to increase the number of periodic Schiff staining (PAS) positive cells and mucin secretion (75,76). They were also tested in a rabbit model of desiccating stress which involved removal of the nictitating membrane, and induction of desiccating stress through the use of constant air flow at a speed of 10m/s applied to the cornea for 10 mins (76). This induced acute corneal damage and provided a model to evaluate protective effects of the treatment. This model will not be suitable to evaluate effect of drugs on existing disease as this a very acute model. Another rabbit model that was utilized to evaluate rebamipide was the model created by instilling 10% N-acetylcystiene (NAC) solutions, a well-known mucolytic and anti-collagenolytic agent into rabbit eyes (77). Treatment with NAC reduces conjunctival mucin levels and hence this model can be useful to evaluate effect of drugs on mucin secretion. A similar model of chemical-induced damage in rabbits has been described which involves the application of the topical antiseptic preservative benzalkonium chloride (BAC) to the ocular surface (reviewed in (78)). BAC-induced dry eye has been reproduced in rodents, rabbits and dogs and has been shown to mimic many signs of human DED (79). BAC has been shown to destabilize tear films, promote overexpression of inflammatory mediators, hyperemia and death of goblet cells. This model has been used to evaluate the therapeutic efficacy of anti-inflammatory steroid and serum eye drops, which is a novel approach in treatment of dry eye (80–82).

Another mechanism that has been explored clinically as a potential secretagogue is modulation of nerve growth factor (NGF) signaling on the ocular surface via the use of the NGF mimetic molecule Tavilermide (MIM-D3, Mimetogen) (83). NGF has shown to stimulate mucin secretion in human conjunctival cells and also in a surgically-induced dry eye dog model (84,85). Surgical removal of the prolapsed lacrimal gland of the third eyelid in both eyes at the age of 3–6 months were shown to develop chronic dry eye with a decrease of Schirmer tears test values. In addition to NGF, topical 2% CsA has also been shown to promote corneal healing and conjunctival goblet cell mucin production in these dogs. Tavilermide is a proteolytically stable, cyclic NGF peptidomimetic partial agonist of the TrkA receptor and has been evaluated in a rat model of dry eye (86). In this study tear deficiency was induced by using scopalmine in rats (87) that resulted in reduced tear production, increased NGF levels in tears and worsening of ocular signs including corneal staining. Topical treatment with 1% Tavilermide resulted in decrease in corneal staining and increases in tear glycoconjugate levels. FDA recently approved the topical recombinant human NGF eye drops Cenegermin for treatment of the rare ocular disorder Neurotrophic Keratitis (88). These observations indicate that a variety of aqueous tear deficiency models have been utilized to evaluate different secretagogues and no single model is sufficient to capture the multifactorial pathology of human DED.

##### Therapies for Ocular Pain and Discomfort

Ocular pain, discomfort and itch are refractory symptoms of many ocular diseases which include dry eye, infectious diseases, allergic conditions and ocular injury. These symptoms are triggered from pathological processes of the disease affecting the specialized nerves of the trigeminal ganglia that express different sensory receptors including the polymodal nociceptors, and the cold thermoreceptors (89,90). The cold thermoreceptor neurons express sensory receptors that play a role in thermoregulation of the ocular surface critical for the maintenance of the tear film via modulation of basal tearing and blinking rates (91). Transient Receptor Potential Melastatin 8 (TRPM8) has been shown as the primary sensory receptor that controls basal tearing as well as eye blinking and may underlie the mechanism by which stimulation with menthol reduces discomfort in patients with dry eye disease by menthol-containing artificial tear eye drops (92–94). Multiple agonists of TRPM8 receptor have been reported to improve ocular surface wetness in the models of tear deficiency described in the previous section above such as those induced with scopalmine and lacrimectomy in rats, mice and even guinea pigs (95–97). While the characterization of signs of dry eye such as neuronal control of tear secretion or blinking can be evaluated in preclinical models, the subjective evaluation of other modalities of pain that are evaluated in humans cannot be easily assessed in preclinical animal models. In animal models, assessments of pain and discomfort must rely on interpretation of observation of animal behaviors associated with pain which can lead to subjectivity and variability of results adding to the challenge of drug development for ocular pain and discomfort. The physiological changes in the function of the sensory neurons have been evaluated in preclinical studies via electrophysiological recordings, which cannot unfortunately be performed in human studies (98,99).

The polymodal nociceptors innervating the ocular surface are activated by noxious stimuli such as strong evaporation, high osmolarity, irritants, injury, inflammation etc. to evoke unpleasant sensations of dryness, blinking, tearing and pain on the ocular surface. Transient receptor potential cation channel vanilloid 1 (TRPV1) is a key receptor for sensory transduction in polymodal nociceptors and has shown to be activated by hyperosmolarity, corneal damage, inflammatory mediators etc., which are present in ocular surface diseases such as dry eye (100). Based on this observation Sylentis is currently evaluating a topical treatment with small interfering oligonucleotide of RNA (SiRNA) targeted to block the human TRPV1, Tivanisran, in Phase 3 studies in dry eye patients (101). One model of acute ocular pain that was utilized to evaluate this molecule is the capsaicin-induced ocular pain model in rabbits (102). It has been shown in this model that topical application of capsaicin, a TRPV1 agonist results in immediate sensations of pain as evidenced by lid squeezing, vocalization, ocular irritation including conjunctival edema and hyperemia. Latency to open the eyes and degree of palpebral opening are used as measures to assess responses to treatment. In these studies, it has been described that Tivanisran showed similar effects to capsazepine which is a TRPV1 antagonist. However, this model has inherent limitations in that it is an acute model of pain induced by a strong noxious stimulus with subjective all or none evaluations without the sensitivity to detect dose responses. Other efforts have been made to use different dry eye models described above to evaluate behaviors such as spontaneous eye blink or eye wiping behaviors as markers of ocular discomfort (73,103–106). In addition, evoked eye blinking and eye wiping responses to various stimuli including hypertonic saline, capsaicin, mustard oil, mechanical stimuli etc. have also been reported. However, the utility of these models in drug development for ocular pain is unclear due to variability and unclear translatability of the specific behaviors to human symptoms. Hence evaluation of drugs targeted to address unmet needs in ocular discomfort and pain may have to be directly addressed in clinical studies.

##### Considerations for Preclinical Models of Ocular Surface Diseases

Summarized in this section are several preclinical models of ocular surface disease that have been utilized in drug development. In the setting of ocular allergy, the preclinical models have shown good predictive value as similar methods of allergic induction are used in human studies. However, these models do not sufficiently address mechanisms of chronic allergy, nor clinical signs observed in severe allergic conditions such as VKC and AKC involving cornea damage. Likewise, the preclinical models for DED do not fully represent the complexities of disease. DED is a multifactorial disease of the tears and ocular surface that results in clinical signs of tear defects, inflammatory mechanisms, and damage to the ocular surface. The etiology of the clinical signs observed in DED can arise from a variety of pathogenic mechanisms that include hyperosmolarity, chronic inflammation, tear deficiency, and damage to ocular surface tissues including epithelium, nerves and secretory glands. No one preclinical model is sufficient to capture all these pathogenic mechanisms. For instance, the lacrimectomy models may be better suited to evaluate drugs aimed at correcting tear defects. While the desiccating stress model in rodents appears to capture the inflammatory mechanisms seen in human dry eye, it is limited by the size of the eye which results in significant differences in pharmacokinetics of the drug and required dosages compared to humans. Furthermore, most of the DED preclinical models are acute in nature, and considerable resources may be needed to house animals for the duration needed to evaluate the true chronicity of DED in a tractable manner in an experimental setting. Another challenge of preclinical models is the inability to directly translate the symptoms observed in a clinical setting including ocular pain, discomfort, visual disturbances etc. This problem is central to the unmet need in the treatment of DED. The use of animal behaviors as a translational representation of human ocular discomfort and pain is still under development and are awaiting validation. Significant progress needs to be achieved to develop additional preclinical models of ocular pain that captures the pathogenic mechanisms of pain in DED. Therefore, currently multiple animal models may have to be utilized for characterizing and evaluating therapies for ocular surface diseases. The features of models that have been used in translation of therapies for these maladies are summarized in Table I.

**Table I:** Preclinical Models Used to Establish POC for Ocular Surface Disease

Model	Species	Pros	Cons	Utility
Histamine-induction of allergic reaction	Dunkin Hartely guinea pig, Wistar rat, Beagle dog	Rapid induction of edema and vascular permeability Amendable to intra-dermal, nasal, plantar, etc. routes of administration	Short duration of effect Does not capture chronic allergy Does not capture keratitis/ulcer	Olopatadine, Emedastine, Bilastine, Cetririzine, Loratadine
Compound 48/80-induction of allergic reaction	New Zealand White rabbit, guinea pig	Rapid histamine-driven lid edema, chemosis, ocular redness, tearing Repeat challenge option	Short duration of effect Does not capture chronic allergy Does not capture keratitis/ulcers	Alcaftadine, Levocabstine
Allergic Conjunctivitis: Ragweed pollen or OVA antigen	Balb/c mouse guinea pig Wistar rat	Rapid induction of lid edema, chemosis, ocular redness, tearing/discharge, scratching/wiping response, vascular permeability Opportunity for longer treatment paradigm Environmental antigen is clinically relevant	Does not capture late-phase response Does not capture keratitis/ulcers Small ocular surface presents differences in pharmacokinetics of the drug and required dosages compared to humans	Olopatadine, Azelastine, Levocabastine, Epinastine, Cetirizine, ST266, Bilastine
Allergic Conjunctivitis: Allergic Eye Disease with OVA antigen	C57Bl/6 mouse	Rapid induction of ocular allergy signs/symptoms, plus meibomian gland plugging, conjunctival fibrosis, eczema of the eyelid	Observed cornea pathology is not a readily translatable clinical readout	EBI-005
		Captures late-phase response, severity of chronic disease with cornea lymphangiogenesis Opportunity for longer treatment paradigm	Small ocular surface presents differences in pharmacokinetics of the drug and required dosages compared to humans	
Desiccating stress model ± scopolamine, atropoine sulfate	C57BL/6 mouse, female	Captures altered tear volume and clearance, corneal barrier function, conjunctival morphology and health, goblet cell density, and immune infiltrate observed in clinical DED Well defined inflammatory mediators observed in clinical DED	Small ocular surface presents differences in pharmacokinetics of the drug and required dosages compared to humans	EBI-005, Tofacitinib
Canine KCS	Beagle, mixed dog	Captures altered tear volume and clearance, conjunctival morphology and health, mucin production, and immune infiltrate observed in clinical DED Large ocular surface is appropriate for translational pharmacokinetic/pharmacodynamic relationship with human	More severe signs; thick ocular discharge, hyperemic conjunctiva, mucous filaments, corneal vascularization, etc. compared to human DED Corneal fluorescein stain readout may not translate to clinical development measures May be difficult to acquire colony with spontaneous disease or enroll patients/pet	Cyclosporine, Lifitegrast
EIU	Lewis rat	Rapid induction of innate cell mediated inflammation Surrogate for DED associated inflammation and amendable to topical drug delivery	Short duration of effect Does not capture T cell mediated inflammation	TOP1362
Excision of Lacrimal Gland	C57BL6, C3H, BALB/c mouse, Wistar, Sprague Dawley rat, New Zealand White rabbit	Rapid induction of tear deficiency Disruption of corneal epithelial barrier Opportunity for longer treatment paradigm Potential to differentiate between anti-inflammatory and other mechanisms of actions	Variability of effects due to surgical and species differences Does not capture T cell mediated inflammation or other deficiencies in the lacrimal functional unit	Diquafasol, Rebamipide, P-321

### Anterior Segment Diseases

Posterior to the cornea lies the remaining portion of the anterior segment which encompasses all the structures between the iris and the lens. The iris is a thin, circular structure which regulates the the pupil diameter size and subsequently the amount of light reaching the retina. Within the space between the iris and lens are critical structures and fluid such as the ciliary body, aqueous humor (AqH), trabecular meshwork (TM). AqH is defined as a thin, clear fluid filling the anterior chamber which is primarily composed of water (99.9%) and trace amounts of sugars, vitamins, proteins and other nutrients as well as growth factors and cytokines. The AqH, along with other structures of the anterior segment work together to support ocular health via management of intraocular pressure, nutritional support to the cornea and lens, and physical support to maintain the shape of the eye. The ciliary body and its muscles modify the curvature of the lens and its ability to focus images onto the retina. Extracellular matrix deposition at the TM leads to increased resistance to aqueous outflow potentially leading to ocular hypertension (OHT). OHT is a risk factor for the progression of glaucoma which is one of the leading causes of irreversible blindness. It is projected that by 2020, nearly 80 million people will suffer from glaucoma worldwide (107). Glaucoma refers to a group of eye disorders, with or without elevated intraocular pressure (IOP), that are characterized by optic neuropathy that includes loss of retinal ganglion cells (RGCs) and their axons at the level of the lamina cribrosa. This in turn leads to the cupping of the optic disc and progressive loss of vision. Glaucoma can be further divided into multiple subtypes including primary open angle (POAG), primary angle-closure (PACG), normal tension (NTG), congenital, exfoliation (or pseudoexfoliation) and secondary glaucoma. The most prevalent types are POAG and PACG, which are characterized by a pathological rise in IOP (107–115).

Hardening of the lens, loss of accommodation and opacification of the lens can lead to presbyopia and cataract (108,116–118). Multiple animal models have been established to mimic the pathology observed in these conditions and in some cases have resulted in approval of therapeutics. In this section of the review we describe animal models that have led to the identification of IOP lowering drugs for glaucoma and end by briefly discussing some of the challenges facing drug development for presbyopia.

#### IOP Lowering Drugs

The maintenance of normal IOP is a well-choreographed interaction of multiple structures in the eye which include ciliary muscles, TM, Schlemm’s canal (SC),, and episcleral veins. AqH drains from the eye via one of two pathways; the conventional TM pathway, or unconventional uveoscleral pathway. Multiple factors can interfere with this process and by doing so inhibit outflow facility which leads to a pathological rise in IOP. Age, race, genetics, oxidative stress, trophic factor dysfunction, excessive extracellular matrix (ECM) deposition, and excitotoxicity are only a few of the other factors described in the literature contributing to glaucoma which have become a focal point for development and selection of animal models (110,119). Currently, the only available treatment for management of this disease are IOP lowering drugs. As a result, preclinical models have focused on decreasing IOP in experimentally induced hypertensive or normotensive models. In this review, we will describe approved IOP lowering treatments and the various preclinical animal models in multiple species that supported their regulatory approvals (see Table II). (120).

**Table II:** Preclinical Models Used to Establish POC for Glaucoma

Model	Species	Pros	Cons	Utility
Normotensive	C57BL6 and Transgenic mouse (e.g. GLAST^−/−^), New Zealand White rabbit, Cynomolgus, Formosan Rock monkey, Beagle dog	RGC death and preservation of RGC’s can be evaluated in the absence of elevated IOP mimicking normotensive glaucoma in humans Higher species have larger eyes and tend to have more similar anatomy to humans	Higher species are more costly and difficult to work with Anatomical differences with humans (e.g. Schlemm’s canal is absent in dogs)	Brimonidine tartrate, Bimatoprost, Netarsudil
Laser Photo-coagulation	Wistar rat (350-450g), C57BL6 mouse, CD1 mouse, New Zealand White rabbit, Young adult Cynomolgus & Rhesus monkey	Reliably induces elevated IOP, degeneration of Optic Nerve and RGC’s	Variability in IOP magnitude and rate of return to baseline	Brimonidine tartrate, Dozolamide, Bimatoprost, Latanprosten bunod, Memantine
		Multi-species adaptable Can mimic POAG or PACG	Potential for retinal edema and hemorrhage	
		in humans depending on target region	Lamina cribrosa absent in mouse	
Steroid-induced Ocular Hypertension	C57BL6 mouse, Wistar and Sprague Dawley rat, rabbit	Inexpensive and non-invasive	Anatomical differences to humans (e.g. lamina cribrosa absent in mouse)	Latanoprost, Pilocarpine, Timolol maleate, Dorzolamide
		Easy to establish Mimics POAG in humans	Prolonged use of steroids can result in cataracts and corneal ulcers	
Water Loading	NZW rabbit	Short in duration and suitable for drug screening Results in RGC loss, decreased aqueous outflow facility	Elevated IOP is short in duration and not representative of POAG	Brimonidine tartrate, Carbonic anhydrase inhibitors (e.g. dorzolamide hydrochloride)
		Mimics PACG in humans	Selective loss of RGCs and broader overall ocular damage	
Hypertonic Saline	New Zealand White rabbit, Brown Norway rat	Single injection reliably induces elevated IOP and can be sustained over a longer period of time with multiple injections	Technically challenging, especially in smaller species and may require multiple injections	Latanoprostene bunod
		Mimics PACG in humans and obstruction of aqeous outflow		
α-chymotrypsin	Albino Dutch Belt and New Zealand rabbit	Longer duration of elevated IOP than hypertonic saline model	Similar results to steroid induced models but in higher species with higher cost	Dorzolamide hydrocholoride, Timolol maleate, Dorzolamide/Timolol maleate Combo
		Elevation of IOP due to trabecular meshwork blockage Mimics POAG in humans	Injection into posterior chamber can result in damage to other structures in the eye	
Microbead/ Microsphere	C57BL6 mouse, Wistar, Brown Norway, Sprague Dawley rat, Macaque	Long duration of elevated IOP observed with single injection in mice	Degree of damage varies between species	Timolol maleate, Brinzolamide
		Multi-species adaptable	Additional variability introduced with acute IOP spikes and differences in induction techniques	
		Mimics PACG in humans with significant loss of RGCs	Microbeads may obscure visibility of optic disc Methods can be technically challenging and may require multiple injections	
DBA/2J Mouse & Other Transgenic Models	DBA/2J mouse (pathology begins at 8 months)	Mimics functional & histological pathology in humans	Small eye and anatomical differences compared to humans	Timolol maleate, Dorzolamide hydrochloride, Brimonidine tartrate, Travoprost, Memantine, Nicotinamide
		Genetic manipulation can represent multiple forms of glaucoma that help to elucidate molecular mechanisms and aid in differentiation of molecules or genetic pathways	Pigmentary glaucoma results in POAG but has PACG component in the DBA/2J mouse	
			IOP elevation due to iris pigment dispersion in DBA/2J	
			Transgenic strains can be difficult to obtain commercially	

Currently approved IOP lowering drugs generally fall into one of three categories based on their mechanism of action: 1) AqH production inhibitors, i.e. carbonic anhydrase inhibitors and adrenergics; 2) drugs that increase AqH outflow, i.e. nonselective adrenergic agonists and prostaglandin analogs; and 3) osmotic agents, such as mannitol or glycerin (121,122). Alpha-2 adrenergic agonists reduce IOP by decreasing AqH production and by increasing AqH outflow and have a long history in treating glaucoma. Clonidine was the first alpha adrenergic drug to be marketed for glaucoma nearly 40 years ago, and recently, safer and more selective alpha-2 agonists have been approved such as Alphagan P® (brimonidine tartrate, Allergan) (123–128). Carbonic anhydrase inhibitors such as Azopt® (brinzolamide, Alcon) and Trusopt® (dorzolamide, Merck & Co.), reduce IOP by their capacity to inhibit AqH secretion at the level of the ciliary body (129,130). Combination therapies are also a mainstay of IOP management including Simbrinza® (brimonidine tartrate + brinzolamide, Alcon), Combigan® (0.2% brimonidine tartrate/0.5% timolol maleate, Allergan), Cosopt® and Cosopt PF® (0.5% timolol maleate +2% dorzolamide hydrochloride; ± preservative, Merck & Co.) (131–134). Enhancing AqH drainage and subsequently reducing IOP has also been a very effective strategy for treating glaucoma. Pilocarpine, a cholinergic agonist launched decades ago for open-angle glaucoma, increases AqH outflow in the TM by directly stimulating ciliary muscle contraction. Pilocarpine has been shown to reduce IOP in multiple animal models described in this review. Similarly, prostaglandin analogs, such as Xalatan® (latanprost, Pfizer), Vyzulta® (latanprostene bunod, Bausch + Lomb), Lumigan® (bimatoprost, Allergan), and Travatan® (travoprost, Alcon), reduce ocular hypertension by increasing AqH outflow. Prostaglandin analogs are currently the most widely used treatments and commonly used benchmarks in animal studies (135). Rhopressa® (0.02% netarsudil ophthalmic solution, Aerie Pharmaceuticals), a Rho kinase inhibitor, is the latest addition to marketed glaucoma topical drugs. It is designed to decrease IOP by increasing conventional outflow and possibly decreasing episcleral venous pressure (136).

Normotensive rabbit and monkey models are the most widely used preclinical animal models to demonstrate IOP lowering efficacy of drugs prior to regulatory approval. These normotensive models have supported approval of brimonidine tartrate monotherapies as well as combination therapies with other compound classes such as carbonic anhydrase inhibitors. Normotensive rabbit and monkey models primarily are performed in New Zealand White rabbits and Cynomolgus monkeys. However, the NDA for bimatoprost reports the use of both normotensive beagles and monkeys to demonstrate efficacy.

In addition to evaluations in normal animals, experimentally-induced ocular hypertension animal models such as laser photocoagulation are utilized to damage aqueous outflow pathways and mimic pathology observed in POAG and PACG. Along with increased IOP, scarring of the TM and nearby ocular tissue, pigment dispersion, RGC death, cupping of the optic nerve head and thinning of the RNFL have all been observed in these types of models. Drugs that have demonstrated efficacy in these models lower IOP by increasing outflow of AqH through both the TM and uveoscleral routes despite laser induced damage. Laser photocoagulation of TM, translimbal or episcleral veins has been conducted in rodents, rabbits, and primates (137–139). However, the magnitude and rate at which IOP returns to baseline are variable. Unfortunately, there is also the risk for potential retinal edema and hemorrhaging (140). In spite of these caveats, data from laser-induced ocular hypertension animal models have been used to support preclinical efficacy for brimonidine tartrate (141), dorzolamide (129), bimatoprost (135), and latanprostene bunod (142). Most recently, the approval of latanprostene bunod, a latanoprost acid prodrug covalently bound by an ester linkage to 4-hydroxybutyl nitrate, was supported by data from multiple animal models listed in the NDA, including the laser-induced ocular hypertension monkey model in female cynomolgus monkeys using an argon laser targeted at the TM, as described by Gasterland and Kupfer (138).

Validation of animal models using clinical comparators is key to determining their translatability since this can give very valuable information to conduct comparator studies in the clinic. Gupta *et al*. (2007) produced data to support the validity of three different rabbit models by comparing the effects of current anti-glaucoma medications (pilocarpine, timolol, and latanoprost) (143). Similar to its effects in humans, latanoprost demonstrated the highest peak reduction in IOP and longest duration of action compared to the two other anti-glaucoma drugs in normotensive, steroid-induced ocular hypertension and water loading models in both male and female New Zealand White rabbits. Woodward *et al*. (2004) compared published preclinical and clinical data from bimatoprost that elegantly demonstrate the superiority of bimatoprost over relevant comparators such as latanoprost, travoprost, timolol and the fixed combination of dorzolamide and timolol (144). It is also reported that responses in the monkey to bimatoprost mimic results observed in humans (145–148). These data are excellent examples of translation between preclinical and clinical studies.

The water loading rabbit model is used to mimic PACG in humans and its use dates back to the 1960’s. This model is characterized by a rapid increase in IOP following administration of water (60–100 ml/kg) through an orogastric tube. A 40% increase in IOP can be observed as early as 15 mins with a peak increase at 60 mins post water loading. These types of experimentally induced models of glaucoma that are short in duration are thought to be most suitable for drug screening and have been used to demonstrate the efficacy of drugs currently approved in the treatment of glaucoma. For example, the water loading rabbit model has been used to demonstrate preclinical efficacy of such compounds as brimonidine, and carbonic anhydrase inhibitors dorzolamide hydrochloride and diclofenamide (125,143,149,150).

Steroid induced models of hypertension are inexpensive and non-invasive models which resemble POAG in humans. Steroids such as dexamethasone can be administered by topical instillation or intravitreal (IVT) injection resulting in elevated IOP, ECM deposition, TM thickening, oxidative stress, and RGC death. This model has been established in multiple species (C57BL6 mice, Wister rats, Sprague Dawley (SD) rats and New Zealand White rabbits) and has been used to demonstrate IOP lowing effects of drugs from multiple drug classes such as dorzolamide, timolol, and pilocarpine (151–153).

In addition to the previously described drug classes, Adenosine A1 agonists and Rho kinase inhibitors have recently emerged as potential novel therapeutics for improving aqueous humor drainage via the trabecular meshwork (154). Adenosine A1 agonists, such as trabodenoson (Inotek Pharmaceuticals Corporation/Rocket Pharmaceuticals), significantly lowered IOP and increased outflow facility via the TM (>25%) in normotensive young and aged C57BL6 mice (155–157). Trabodenoson also demonstrated efficacy, safety and tolerability in Phase I and II clinical trials. However, it recently failed in Phase III as a monotherapy and in a Phase II trials as a combination treatment with latanoprost. It was concluded that combination treatment yielded no meaningful clinical advantage over latanoprost monotherapy. Rho kinase (ROCK) inhibitors appear to be the most promising, with the recent approval of Rhopressa® (netarsudil, Aerie Pharmaceuticals). As previously mentioned, netarsudil is thought to lower IOP by increasing outflow through the TM, decreasing AqH production and episcleral venous pressure. With this recent approval, ROCK inhibitors are the first new class of glaucoma medication to be approved in more than 20 years. Preclinically, Aerie Pharmaceuticals demonstrated significant reductions in IOP following netarsudil treatment in normotensive rabbits and monkeys. In normotensive cynomolgus monkeys, IOP was decreased by ~25%, outflow facility increased by 53%, and mean AqH flow rate was reduced by ~20%. Dose-dependent IOP lowering effects were also observed in Dutch-belted rabbits and normotensive Formosan Rock Monkeys (136,155).

Less commonly utilized models leading to regulatory approval of anti-glaucoma medications are the hypertonic saline and α-chymotrypsininduced ocular hypertension models. These models have been induced predominantly in Brown Norway rats and New Zealand rabbits, respectively. To induce elevated IOP in the saline model, injections of hypertonic saline are administered in the anterior chamber or episcleral veins. A single injection or multiple injections can induce sustained and elevated IOP (137,158). IOP lowering effects of latanoprostene bunod were demonstrated using this model via injection of 0.1mL of hypertonic (5%) saline into the anterior chamber of both eyes in New Zealand White rabbits. The α-chymotrypsin induced IOP model increases IOP by blockage of AqH outflow as well as an inflammatory response that may be contributing to sustained IOP observed in this model. To induce chronic elevated IOP, 0.1mL of α-chymotrypsin is injected into the posterior chamber (133,159). This model was used to differentiate between a preservative-containing and a preservative-free dorzolamide hydrochloride-timolol maleate fixed combination.

Additional preclinical models of OHT or glaucoma such as genetic models, and microbead injection have been utilized for proof of concept and mechanistic studies.. Injection of microbeads or microspheres into the anterior chamber is a minimally-challenging, multi-species adaptable model. However, multiple injections may be required to maintain the necessary elevation in IOP. Depending on the species and type of injury, some of these animal models can represent either the POAG or PACG subtypes (109,137,139,140,160–162). Timolol and brinzolamide have been evaluated in microbead induced ocular hypertension models in adult C57BL6 mice and demonstrated reductions in IOP as well as improved RGC and axon survival over controls (163). Performing these models in higher species may increase translatability to humans but they also have their disadvantages. Despite the obvious benefits of similar homology and ocular anatomy, working with primates can be costly and difficult due to ethical limitations and specialized skills and facilities required for primate research (163–165).

Genetic models of glaucoma have been established in multiple species. The DBA/2J mouse strain is the most commonly used and most characterized genetic model of progressive secondary angle-closure glaucoma (166). A PUBMED search of the DBA/2J mouse strain and glaucoma yields over 200 articles spanning just over 20 years. Various mutations and crossings with other strains (e.g. DBA/2NNia, AKXD28) have been used to model transient ocular hypertension with both functional and histological pathology similar to the human disease (167–170). One drawback of the DBA/2J mouse is that the mechanism inducing elevated IOP in this strain is due to iris pigment dispersion and iris stroma atrophy. The specificity of this mechanism may not allow for a broader interpretation of results from this model (140,168). However, differences in susceptibility of ocular hypertension between mice strains can help elucidate the molecular mechanisms, or aid in differentiation of molecules or genetic pathways crucial to the development of the various subtypes of glaucoma (112).

Spontaneous inheritance of glaucoma in dogs provides additional opportunities to both identify genes responsible for glaucomatous pathophysiology as well as evaluate potential treatments. These models have been used to describe multiple subtypes of glaucoma including PACG, POAG, and congenital glaucoma (137,171–173). Mutations in genes such as MYOC, CYP1B1 and ADAMTS10 have been found in humans with POAG and have also been explored in beagles and other dog breeds with inherited glaucoma (174–176). Furthermore, both normotensive and inherited glaucomatous beagle dog models have demonstrated similar findings of reduced IOP with approved antiglaucoma medications such as brimonidine tartrate, timolol, and pilocarpine (177–180). A drawback of naturally occurring models is that animals may not all exhibit onset of disease at the same time and progression of disease is likely to occur at different rates. Additionally, differences in ocular anatomy between species should always be taken into consideration. For example, mice lack collagen bundles found at the lamina cribrosa (LC) which is otherwise conserved in rats, primates and humans and is considered important in the pathophysiology of glaucoma (140,181,182). Other models of interest include transgenic mice overexpressing the optineurin E50K mutation, transgenic TBK1 mice, as well as glutamate transporter knock-out mice (112,183–185).

#### Presbyopia

Presbyopia typically strikes individuals over the age of 40 and is a progressive loss of accommodative function in which near vision is compromised. Accommodation relies on the contraction of the ciliary muscle in harmony with changes in the lens and binocular convergence. Drugs proposed to treat presbyopia attempt to pharmacologically manipulate accommodation via parasympathetic stimulation of the ciliary muscle or the use of miotics to increase depth of focus by decreasing pupil size, also known as the pinhole effect (186). Factors that contribute to presbyopia include hardening of the lens, changes in elasticity of the lens capsule, lens dimension, and abnormal ciliary muscle contraction (118,187). Current treatment options for presbyopes include reading glasses, contacts or intraocular lenses. Despite its high incidence and the attractiveness of a pharmacological alternative for presbyopes, there are no FDA-approved drugs for the treatment of presbyopia and descriptions of *in vivo* animal models of presbyopia in the literature are limited. This is due in part to the different accommodation mechanisms between humans and most other species. Monkeys are the only animal species with similar accommodative mechanisms and therefore the most potentially translatable model (188). Kaufman *et al* (1982) established and characterized a monkey model of presbyopia that mimics age-related changes observed in humans (189). Non-invasive imaging techniques, measurements of refraction, and lens diameter are just a few key endpoints in this model that can be used to assess age-related and pharmacologically induced changes in monkeys. Using this model, Wendt *et al*. (2008) confirmed the previously reported presbyopic changes that are shared between monkeys and human, further substantiating the utility of this model for assessing efficacy of potential presbyopic treatments (190). According to a recent search for presbyopia drugs via Clarivate Analytics Integrity, there are currently 14 drugs under evaluation for the treatment of presbyopia. However, there is limited information on preclinical models used to evaluate these treatments in development.

##### Considerations for Preclinical Models of Anterior Segment Diseases

Reconciling the differences between preclinical and clinical research is a common problem to which ophthalmic diseases of the anterior segment are not immune. Inconsistencies observed between preclinical and clinical trials can be attributed to anatomical differences between species, pathogenesis, and differences in endpoints and overall study design. In glaucoma, there are many published animal models for IOP but only a few that have been consistently utilized for regulatory approval of drugs. Meta-analyses of published data show that primate models of ocular hypertension are most predictive of clinical success (191). Progression of disease in the absence of elevated IOP emphasizes the need to focus on models that can accurately predict the clinical efficacy of potential neuroprotective therapies. Presbyopia animal models face a similar problem in that with no currently approved drugs, it is difficult to assess whether our current models are predictive. Bridging the gap between preclinical and clinical endpoints, focusing on ones that are most indicative of drug efficacy in both domains, will improve translatability.

### Retinal Diseases

The retina constitutes the neurosensory tissue of the eye and is comprised of multiple neuronal layers and a tightly-regulated vascular network. Light entering the eye is detected by rod and cone photoreceptors that synapse with bipolar, horizontal and amacrine cells. These second order neurons connect to ganglion cells whose axons form the nerve fiber layer, which transports the visual signal through the optic nerve to the brain. The exceptionally-high metabolic demands of the retina are served by two vascular beds; the choriocapillaris is a dense network of highly fenestrated capillaries which oxygenates the outer retina and the intra-retinal vasculature is an end-artery, multi-layered capillary network perfusing the inner retina. The most prevalent forms of retinal disease, age-related macular degeneration and diabetic retinopathy, are associated with pathological changes in both neuronal cells of the retina as well as its vascular networks. If untreated, or treated insufficiently, the disrupted supply of nutrients and oxygen to the photoreceptors results in their death, and eventually, vision loss.

Glaucomatous Optic Neuropathy. Glaucomatous optic neuropathy is a complex neurodegenerative disease that affects the RGCs and their subsequent axonal projections leading to the optic nerve. It is characterized by optic nerve head remodeling, RGC death and visual field defects (110,192). Ocular hypertension is a major risk factor for progression of glaucoma that when well-controlled, can slow progression of disease. However, in some cases further deterioration continues despite management of IOP. Neuroprotective strategies and animal models focused on RGC and axonal loss have been employed to address the degeneration observed in glaucoma. (112,193–195). Optic nerve injury models provide the ability to evaluate the pathophysiological events following the initial injury or primary degeneration of axons as well as the opportunity to differentiate between primary and secondary degeneration, and assess neuroprotective potential of drugs (109,125,196). These models are typically conducted in rodents (SD and Wistar rats) but have also been established in primates.

Optic nerve injury models may include transection (partial or complete) or crushing of the optic nerve itself to inflict initial damage to axons and RGC bodies. In a complete optic nerve transection model, all axons are transected and RGC death is inevitable, making it difficult to differentiate between primary and secondary degeneration. Partial optic nerve transection causes damage to a specific set of axons in the optic nerve leading to regionally specific degeneration, based on their projections, to RGC bodies. This model also allows for secondary degeneration of neighboring cells that are distinguishable from the initial injury based on location. The damage observed in the optic nerve crush model resembles that which is seen in the human disease and has been extensively used to support brimonidine in the literature as a treatment for glaucoma. One disadvantage of this model is that the amount of force as well as the duration that force is applied for is not standard and can vary between laboratories. Furthermore, damaged axons are intertwined with surviving ones making it difficult to distinguish between primary and secondary degeneration (109,193,194,196–198). Of these models, the partial optic nerve transection model is assumed to be the best model of secondary degeneration to assess efficacy of neuroprotective test agents.

Genetic models such as GLAST and EAAC1 deficient mice described above (112,184) are also valuable for evaluating neuroprotection strategies in glaucoma. They have the potential to provide a unique opportunity to mimic the pathological effects of naturally progressing glaucoma on RGCs and axons.

Although there are many instances of preclinical models demonstrating neuroprotection, not only for glaucoma, but in a wide range of neuropathies, there is still an unsettling amount of translational failures in clinical trials of neuroprotection. One example of this related to glaucoma is the Memantine in Patients with Chronic Glaucoma Study (NCT00168350) (199). Woldemussie *et al*. (2002) reported evidence of protection of RGCs in two rat glaucoma models with memantine treatment (198). A partial optic nerve injury was performed in SD rats by applying a calibrated amount of pressure to the optic nerve for 30s at approximately 2-3mm from the globe. A single dose of memantine, administered immediately after injury, enhanced survival of RGCs and preserved nerve function, as measured by electrophysiology. In a second rat model, chronic ocular hypertension was induced using laser photocoagulation of the limbal and episcleral veins. Chronic treatment of memantine reduced RGC loss but had no effect on IOP (200). The neuroprotective effects of systemic memantine treatment were also demonstrated in monkeys with laser induced damage to the anterior chamber angle. Memantine treatment enhanced survival of RGCs and reduced multifocal ERG deficits in memantine treated monkeys compared to vehicle (201,202). Atorf *et al*. (2013) also generated animal model data to support neuroprotective effects of memantine treatment in the DBA/2J mouse model of congenital glaucoma (203). Despite these positive results in preclinical studies, memantine treatment did not reduce progression of glaucoma in the human clinical trial. This may have been due to challenges in assessing visual acuity improvements, and translational measurement of visual field loss in animals in a way that is relevant to humans. Another significant issue is the multifactorial nature of optic neuropathy which emphasizes the need for more models to recapitulate these complexities and multiple mechanisms of action, single or in combination, to be addressed in patients.

#### Age-Related Macular Degeneration

Age-related macular degeneration (AMD) is a chronic, multifactorial disease characterized by progressive degeneration of the central region (macula) of the retina, drusen formation, and subsequent atrophy and/or choroidal neovascularization (CNV) (204). It is considered the leading cause of blindness in older individuals in developed countries and estimated to affect ~290 million people by 2040 (205) based on the expanding aging population worldwide. AMD patients report a noticeable decrease in visual function after progressing to late-stage disease, which can manifest in two ways: wet or neovascular AMD (nAMD) wherein new vessels emerging from the choroid penetrate Bruch’s membrane resulting in vascular leakage, hemorrhage, and scarring; and dry or non-neovascular AMD characterized by the presence of drusen, followed by degeneration of photoreceptors, RPE, Bruch’s membrane, and even the choroidal vasculature (206,207). The most advanced stage of AMD, geographic atrophy (GA), is characterized by the development of retinal atrophic areas and eventual permanent loss of visual acuity. Various animal models of AMD have been developed to aid in our understanding of what drives disease progression (208). Histopathological features observed in eyes of AMD patients including changes in RPE, Bruch’s membrane, photoreceptors and CNV have been modeled using mice, rats, pigs, rabbits, and non-human primates (208–210). These models have identified biological pathways involved in disease progression and also serve as useful tools to evaluate novel therapies (211).

#### Neovascular AMD

##### Anti-VEGFs

Vascular endothelial growth factor (VEGF) is an endothelial cell mitogen essential for angiogenic growth of new blood vessels (212). There is a wealth of research associating VEGF expression with CNV in human AMD specimens (213) and also in experimentally-induced CNV in animal models (214–216). Currently, there are three FDA-approved drugs for the treatment of AMD which target VEGF biology: Macugen® (pegaptinib, Bausch + Lomb), Lucentis® (ranibizumab, Genentech), and Eylea® (aflibercept, Regeneron Pharmaceuticals). Besides these, Avastin® (bevacizumab, Genentech), which is a recombinant humanized monoclonal antibody against VEGF, is also used off-label for nAMD. Pegaptinib, an aptamer inhibitor of the pathologic VEGF_165_ isoform, was the first breakthrough therapy to be approved for the treatment of CNV when injected intravitreally (IVT) in patients. Ranibizumab is a recombinant monoclonal antibody fragment (fab) that acts by binding to all active forms of human VEGF and preventing their interaction with the VEGFR1 and VEGFR2 receptors, leading to reduced proliferation, vascular leakage, and neovessel formation. The latest drug to be approved for the treatment of nAMD (2011), aflibercept, is a fusion protein which acts as a decoy receptor for all VEGF isoforms and placental growth factor (PIGF). All approved anti-VEGF agents were tested preclinically in the laser-induced CNV model in non-human primates (NHP), demonstrating its positive predictive value (217). In this model, laser photocoagulation is used to rupture Bruch’s membrane, which causes a local inflammatory response and growth of new blood vessels from the choroid to the subretinal area (218). Intravitreal administration of ranibizumab at 2-weeks intervals prevented the formation of CNV in cynomolgus monkeys on day 21 and repeat administration at 3 weeks and 5 weeks post-laser injury reduced leakage (219). Intravitreal administration of aflibercept in adult cynomolgus monkeys pre-injury prevented the development of CNV, and post-injury, inhibited active CNV leakage (220). Several other proof of concept studies supported the development of aflibercept; for instance, using a mouse CNV model with increased VEGF levels and neovascularization, a single intravitreal injection reduced CNV area. Using a rhodopsin promoter-driven VEGF overexpression transgenic mouse model (rho/VEGF, post-natal 21) with subretinal neovascularization, subcutaneous injections significantly reduced CNV area (221). Collectively, these preclinical studies confirmed that VEGF suppression inhibits experimental CNV, and along with the observed clinical efficacy of anti-VEGF agents, confirmed VEGF’s role in the pathogenesis of nAMD.

Although anti-VEGF therapies are effective in decreasing leakage and halting progression of neovascularization, they come at a cost of high treatment burden for patients, with frequent visits for intraocular injections. Two different strategies are being explored in clinical development which have the potential to decrease patient burden; sustained delivery of anti-VEGFs and injection of long-acting VEGFs. Regenxbio’s RGX-314, a recombinant AAV8 containing a gene encoding for a monoclonal anti-VEGF antibody is currently recruiting for phase I clinical trials. Two key preclinical transgenic mouse models were used to support the progression of this therapy to clinical development; subretinal injection of RGX-314 in rho/VEGF mice showed a dose-dependent suppression of subretinal neovascularization, and in a more severe model of nAMD and retinal detachment, the Tet/Opsin/VEGF mouse model, there was a significant reduction in retinal detachment (222). Adverum Biotechnologies is pursuing a next generation AAV (AAV.7m8) based gene therapy encoding aflibercept, and AGTC is currently developing GZ-402663 (AAV2-sFLT-01), an AAV vector gene therapy that encodes a fusion protein based on the soluble hybrid form of VEGF receptor 1. Both employed the NHP model of laser-induced CNV in cynomolgus monkeys to demonstrate long-term efficacy and tolerability (223,224). Importantly, because experimental laser-induced CNV is self-resolving, to establish long-term efficacy, these gene therapy assets were administered months or more in advance of the model’s induction, in prevention mode.

Sustained delivery of anti-VEGF is being pursued by Genentech with ForSight VISION4’s port delivery system, which is a refillable implant designed to provide sustained release of ranibizumab to the vitreous. The authors could not find any preclinical information linked to this system. Another company pursuing sustained release assets for nAMD is Graybug Vision (GB-102, VEGF/PDGF antagonist, sunitinib, in bioabsorbable microparticles), who employed the laser-induced CNV model in C57BL/6 to establish duration of efficacy (225). The long-term safety and efficacy of sustained VEGF suppression in nAMD patients will be determined during clinical trials and, if launched, in the context of real-world patient observations.

##### Drugs Acting on Other Angiogenesis Targets

Despite the effectiveness of anti-VEGF monotherapy, gaps exist in the treatment of nAMD including but not limited to treatment resistance and marginal durability. To overcome these challenges, drug developers have turned to additional targets in angiogenesis pathways. A primary underlying cause of CNV pathogenesis is changes in pro- and anti-angiogenic growth factors derived from RPE. Pro-angiogenic factors, such as VEGF, fibroblast growth factor (FGF), and platelet derived growth factor (PDGF), are known to stimulate endothelial cell proliferation in CNV (226). Several POC studies were carried out in different experimental CNV models demonstrating that targeting PDGF in combination with VEGF could potentially reduce pericyte-like scaffold formation (hypothesized to support the infiltration of neovessels) and help in the attenuation of CNV via a disease modification mechanism of action, namely, vessel regression (227,228).

Opthotech’s pegpleranib sodium (Fovista®) is a 32-mer pegylated anti-PDGF aptamer hypothesized to increase neovascular membrane susceptibility to VEGF inhibition by stripping vascular support cells, pericytes, from the endothelium. It was found to be more effective in combination with an anti-VEGF agent compared to either PDGF or VEGF inhibition alone in the prevention and regression of pathological vessel formation in a murine laser-induced CNV model generated using male C57 BL/6J mice (228,229). Regeneron tested the effects of blocking PDGFb/ PDGFR-b signaling using an antibody, rinucumab, in a C57Bl/6 mouse model expressing humanized PDGFR-b, which resulted in loss of retinal pericytes (230). OHR pharmaceutical’s ophthalmic formulation, squalamine lactate, is a tyrosine kinase inhibitor, acting on multiple growth factors (VEGF, PDGF, and FGF) which regressed CNV in laser-induced CNV induced in adult Brown Norway rats (231) and reduced iris neovascularization in a laser-induced CNV cynomolgus monkey model (232), but failed to show long term reduction in patients with weekly IV infusions. Despite positive preclinical efficacy, these anti-PDGF strategies did not show differentiation compared to anti-VEGF therapies alone (233). Lack of translation of preclinical to clinical efficacy may in part be attributable to differences in primary endpoints; in preclinical models, emphasis was placed on anatomical changes, such as vessel regression, whereas change from baseline BCVA was the primary endpoint used in clinical trials.

The Tie2 receptor is predominantly expressed on vascular endothelial cells. Angiopoietin-1 (Ang1) is its natural agonist ligand, and angiopoietin-2 (Ang2) is a context-dependent antagonist implicated in nAMD and pathological neovascularization. Several studies have shown elevated levels of Ang2 in nAMD patients (234–236) and one study reported a genetic association with Ang2 SNPs (237). Preclinical studies of Ang2 overexpression show vascular de-stabilization, pericyte drop-out and neovascularization (238,239). Two compounds targeting Ang2 have been evaluated in the clinic: Regeneron’s nesvacumab co-developed with Bayer, and Roche’s RG7716. Nesvacumab, in combination with aflibercept, failed to show superiority in a Phase 2 trial for nAMD compared to aflibercept monotherapy. Two preclinical studies supported its progression to the clinic; one in a rabbit model of chronic vascular leak and another in a Matrigel-induced CNV model in SD rats in which subcutaneous administration of nesvacumab significantly inhibited CNV lesion formation (240). The rabbit chronic vascular leak model is induced by intravitreal injection of a gliotoxin, DL-alpha-aminoadipic acid (AAA), that causes disruption of the blood-retina barrier secondary to toxicity to glial cells in the retina and retinal degeneration (241,242). This disruption is followed by pathological neovascular tuft formation and vascular leakage that persists for months, making it suitable for longitudinal studies assessing duration of action. Using 8–10 weeks old male New Zealand White rabbits injected IVT with dlAAA, Regeneron showed near complete leak suppression with the combination treatment of nesvacumab and aflibercept for an additional 2 to 3 weeks compared to aflibercept monotherapy (243), and there was evidence of blood vessel remodeling after 20 weeks of treatment (244). Roche is developing a novel bispecific antibody, RG7716, targeting VEGF and Ang2 using a novel CrossMab technology and has completed a phase 2 study. Results from this study demonstrated sustained vision outcomes for patients treated every 16 weeks or 12 weeks relative to ranibizumab-treated patients dosed every 4 weeks over a 52-weeks period. Two preclinical studies have been described in support of its clinical program; one in an NHP CNV model using cynomolgus monkeys in which it was shown to suppress lesion formation more effectively than anti-VEGF treatment alone (245) and the other in a spontaneous CNV model in JR5558 mice (described in further detail in the DR section below) in which it was shown to decrease lesion number, vascular permeability, retinal edema, and neuronal loss more effectively than anti-VEGF alone (246). These results suggest a key role for targeting the Tie2 pathway in nAMD via inhibition of Ang-2 mediated vessel leakiness in addition to VEGF-mediated neovascularization.

Neovascular AMD develops because of the formation of a CNV lesion, which can progress to become fibrotic and form disciform scars (204). Despite the improved visual outcomes seen with anti-VEGF therapy, roughly half of patients receiving this treatment still develop subretinal fibrosis within 2 years of starting treatment (247). The laser-induced CNV mouse model has been used recently as a model of subretinal fibrosis (248). CNV develops early in this model (day 7) followed by its regression and complete disappearance after 35 days, but fibrosis continues to develop to 35 days post-induction and beyond. This is consistent with observations in nAMD patients who develop fibrotic scars after CNV. Additionally, RPE in the subretinal lesions in these mice trans-differentiate into myofibroblasts, which are also found in excised human fibrotic membrane from nAMD patients (213). This aspect of the laser-induced CNV model could serve as a great tool for future therapeutic strategies aimed at preventing or regressing subretinal fibrosis.

#### Non-exudative AMD and Geographic Atrophy

##### Neuroprotectants and Anti-Oxidants

The retina is very prone to oxidative injury due to a high concentration of oxidation-susceptible polyunsaturated fatty acids in the outer segments of photoreceptors, high metabolic demand, and the presence of photosensitive molecules like rhodopsin and lipofuscin, which, when exposed to light, produce reactive oxygen species. Oxidative damage has been linked with the development and progression of AMD (249,250), and several preclinical models, mostly knockout mice, have been developed to understand its role in AMD pathology, although none of these genes have been linked to AMD (210). A light-induced photoreceptor degeneration rodent model using 6–7 weeks old SD rats (251) was used to support the development of OT-551 (Othera Pharmaceuticals Inc.), a superoxide dismutase mimetic, which has been evaluated in GA because of its anti-oxidant properties. Intraperitoneal administration of OT-551 was shown to provide both preserve functional and morphological RPE and photoreceptor properties following acute light-induced damage (252). Despite positive preclinical data, the drug failed to slow GA progression in a phase 2 study when administered via eye drops.

Neuroprotective agents may preserve macular function by preventing RPE and photoreceptor cell death. Brimonidine tartrate (Allergan plc), an alpha-2 adrenergic receptor agonist, has been tested in the clinic as an intravitreal implant for the treatment of GA associated with advanced AMD. It is already approved for the treatment of open-angle glaucoma and has been evaluated in many experimental models for its neuroprotective effects, as described in a review by Saylor *et al*. (253). A key preclinical study supporting its development as a potential drug for GA was performed in an NHP model of GA, developed by inducing lesion formation in female cynomolgus monkeys using blue light irradiation (55). Progressive RPE loss was demonstrated by quantification of hypofluorescence. Photoreceptor structure and function were evaluated using optical coherence tomography (OCT) to measure outer nuclear layer (ONL) thickness and multifocal electroretinography (mfERG), respectively. Results showed that the brimonidine implant slowed the increase of hypofluorescence, increased ONL thickness, and reduced mfERG b-wave, suggestive of neuroprotective effects in the RPE and photoreceptors (254). Phase 2a clinical data indicate that brimonidine slowed lesion growth in GA patients (255) similar to preclinical data in NHPs, which utilized endpoints similar to those used in the clinic.

##### Visual Cycle Modulators

Visual cycle biproducts have been implicated in the pathogenesis of AMD via observations of the accumulation of lipofuscin pigments, which consist of a fluorophore called *N*-retinyledin-*N*-retinylethanolamin (A2E) known to be cytotoxic to RPE cells and photoreceptors. The synthesis of these pigments is mediated by retinol influx into the RPE from the serum, which is driven by the formation of retinol-binding protein (RBP4)-transthyretin-retinol complex. Based on this biology, several compounds have been tested in the clinic to reduce serum retinol levels with the goal of decreasing A2E levels. Fenretinide, an oral RBP4 antagonist, developed by ReVision Therapeutics, failed to halt the growth of GA lesions in a phase 2 study. POC studies were carried out in ABC4−/− mice, which have increased accumulation of A2E in photoreceptors and RPE eventually leading to their degeneration. Fenretinide reduced the accumulation of A2E in the RPE by lowering serum retinol levels (256). ACU-4429 or emixustat hydrochloride (Acucela Inc.), an RPE65 isomerase inhibitor, showed a reduction in A2E levels when administered orally in ABC4/RDH8 double knockout mice, a model that not only shows retinal A2E accumulation but also RPE and photoreceptor dystrophy (257,258). However, it also eventually failed in Phase 2b/3, as it did not meet its primary endpoint of reduced GA lesion growth compared to placebo. It is possible that the importance of A2E in AMD pathophysiology was overestimated as demonstrated by poor predictability between lipofuscin accumulation and GA lesion spread in AMD patients (259), differential spatial correlation between A2E and lipofuscin in human *versus* mouse RPE (260) and more importantly, a poor correlation between A2E and lipofuscin in human RPE (260).

##### Complement Inhibitors

Research over the last decade has identified the role of inflammation, more specifically the complement pathway, in the pathogenesis and progression of advanced AMD with several complement proteins detected in the drusen of AMD patients (261,262). Additionally, studies have associated genetic variants and chronic activation of the alternative complement pathway with the development of GA (263). Activation of the complement cascade engages systemic immune cells, hypothesized to cause cell death in the macula. Several therapeutic agents targeting the complement system have been tested in the clinic.

Eculizumab (Alexion Pharmaceuticals) is a humanized murine C5 antibody that binds to human complement factor 5 and prevents its pro-inflammatory cleavage to C5a which mediates inflammation, cellular migration and cytokine release. C5b plays a role in the formation of membrane attack complex (C5-b9), which initiates cell activation and lysis. C5 and its fragments are not only components of drusen in AMD patients (264) but have also been found to be upregulated in the serum of AMD patients (265). Based on this observation, eculizamab was administered intravenously in clinical trials in GA patients and despite showing systemic complement inhibition, it failed to have any effect on GA progression. The POC study supporting the development profile was carried out in an experimental autoimmune uveoretinitis model using an anti-mouse C5 monoclonal antibody, where it was found to suppress myeloid activation and, consequently, retinal tissue damage when administered both systemically and intravitreally (266). Another C5 targeting molecule tested in the clinic is an aptamer, avacincaptad pegol sodium (Zimura®, Ophthotech Corporation) with the same rationale as eculizumab. A phase 2/3 clinical trial is underway evaluating the intravitreal safety and efficacy in patients with GA. The preclinical study supporting its development was performed in a retinal degeneration mouse model of advanced AMD having a mutation in either monocyte chemoattractant protein 1 (MCP-1 or CCL-2) or its cognate receptor (chemokine receptor 2, CCR2) and presenting with drusen below the RPE, accumulation of lipofuscin in RPE, photoreceptor atrophy, and CNV. Furthermore, it displays increased accumulation of C5 in the RPE and choroid (267).

Lampalizumab (Roche), a humanized Fab fragment against complement factor D, failed in two pivotal phase 3 trials (268). Complement factor D (CFD) is the rate limiting enzyme in the alternate pathway (AP) and is present at relatively low plasma concentrations relative to other complement factors in AMD patients. This was the basis of inhibiting CFD locally by testing intravitreal lampalizumab in both patients and preclinically in adult male cynomolgus monkeys in which it was shown to only transiently inhibit systemic AP activity (269). Preclinical and clinical data for molecules targeting the complement pathway showed systemic inhibition, indicating that the molecule was able to bind to and neutralize the target despite not influencing disease progression in the eye.

Apellis Pharmaceuticals is developing a complement C3 inhibitor (APL-2/AL-78898A) for nAMD and GA, which prevents downstream complement activation in all three complement activation pathways. In a phase 2 trial for GA, APL-2 administered monthly showed a 29% reduction in the rate of GA lesion growth compared to sham after 12 months of treatment. The activity of APL-2 in the prevention of complement activation was evaluated in a light-induced injury model using cynomolgus monkeys. The authors reported a significant reduction of C3a and in C5b-9 levels in the retina and RPE/choroid (270). These studies illustrate the challenges of using preclinical models to establish POC for molecules targeting the complement system, despite the significant association of AMD with genetic variations of several complement genes. It is of note that positive clinical data were obtained by Apellis, who demonstrated local target engagement and a significant effect on downstream membrane attack complex levels in their preclinical studies prior to entry into clinical development. Several animal models recapitulating complex pathological features of AMD with their pros and cons are illustrated in Tables III and IV.

**Table III:** Preclinical Models Used to Establish POC for Neovascular AMD

Model	Species	Pros	Cons	Utility
Laser-induced CNV	C57BL6 mouse, Cynomolgus monkey, Brown Norway rat	Anti-VEGF responsive Large eye versions facilitate intraocular dosing/PK-PD correlation	Intra-operator variability in model induction	Aflibercept, Ranibizumab, Pegaptinib, GZ-402663, ADVM-022, GB-102, Pegpleranib Sodium, Squalamine lactate, Rinucumab
		Demonstrated ability to differentiate non-VEGF inhibitors from anti-VEGF	Acute/self-resolving injury model	
		Ability to quantify leak, neovessel formation and vessel regression	Technical challenges with intraocular dosing in lower species	
Matrigel-induced CNV	Sprague-Dawley rat	Demonstrated ability to differentiate non-VEGF inhibitors from anti-VEGF	Small eye limits ability to test intraocular dosing	Nesvacumab
		Longer injury duration (*vs*. laser-induced CNV) Anti-VEGF responsive Ability to quantify leak and neovessel formation	Variability in observed CNV pathology	
Spontaneous CNV	JR5558 mouse	VEGF-driven leak and lesion growth Neovascularization from choroid	Small eye limits ability to test intraocular dosing	RG7716 (faricimab)
		Non-injury model Anti-VEGF responsive	Difficult to establish anti-VEGF differentiation	
Transgenic	Rho/ VEGF, Tet/ Opsin/ VEGF mouse	Anti-VEGF responsive Ability to control VEGF expression allows testing of sustained-delivery anti-VEGF strategies	VEGF-driven does not capture additional pathogenic factors Retinal and choroidal neovascularization observed	Aflibercept
		Ability to quantify CNV area	Small eye limits ability to test intraocular dosing	
			No demonstrated ability to quantify leak and vessel regression	
dl-AAA-induced persistent retinal vascular leak	Dutch Belt/ New Zealand White rabbit	Chronic model allows for longitudinal studies assessing duration of action	Difficult to establish anti-VEGF differentiation	Nesvacumab
		Demonstrated ability to quantify leak, neovessel formation and vascular regression	Anatomical difference between rabbits and humans Doesn’t capture all	
		Large eye facilitates intraocular dosing/PK-PD correlation	relevant phenotypes

**Table IV:** Preclinical Models Used to Establish POC for Retinal Degeneration

Model	Species	Pros	Cons	Utility
Acute Light-induced photoreceptor degeneration	Sprague-Dawley rat, Cynomolgus monkey	Retinal damage caused by oxidative stress Relevant model to explore neuroprotection strategies	Rapid degeneration necessitates prevention design to show therapeutic effect Lack of progression may not capture mechanisms relevant to human disease	OT-551, APL-2
Progressive light-induced photoreceptor degeneration	Cynomolgus monkey	Chronic, progressive model Loss of RPE function precedes photoreceptor death *In vivo* measurement of clinically-relevant endpoints Large eye facilitates intraocular dosing/PK-PD correlations	Ethical and logistic concerns, especially for earliest discovery efforts	Brimonidine tartrate
Genetic models	ABCA4−/− mouse	Lipofuscin and A2E accumulation in the RPE RPE dystrophy Mutation linked with a high risk of AMD in humans	Small eye limits ability to test intraocular dosing Limited utility as this model is specific to A2E biology Different correlation between spatial distribution of A2E and lipofuscin as compared to human RPE	Fenretinide
	ABCA4/RDH8 double knockout mouse	A2E accumulation in the RPE RPE and photoreceptor dystrophy Mutations linked with a high risk of AMD in humans	Small eye limits ability to test intraocular dosing Limited utility as this model is specific to A2E biology	Emixustat
	Ccl2−/− and Ccr2−/− mouse	Degeneration driven by defective complement system Accumulation of lipofuscin in RPE and photoreceptor	Lengthy induction time Variable reports of drusen-like deposits Small eye limits ability to test intraocular dosing	Avacincaptad Pegol Sodium
Autoimmune uveoretinitis	Mouse	Structural retinal damage mediated by infiltrating macrophages Relevant model for testing effect on local complement activation	Small eye limits ability to test intraocular dosing Does not directly capture specific GA-like phenotypes	Eculizumab
Optic Nerve Injury	Sprague Dawley, Wistar rat	Multi-species adaptable and mimic loss of RGCs and axons Partial injury models may distinguish between primary and secondary degeneration Complete injury	Not all models address secondary degeneration May have selective loss of RGCs and axons	Alpha2 adrenoreceptor agonists (e.g Brimonidine tartrate)
		models are easy to establish and yield consistent results	Partial injury methods are technically challenging and vary between laboratories	

#### Diabetic Retinopathy and Diabetic Macular Edema

In the retina, endothelial cells, pericytes and astrocytes work in coordination to form a physical and chemical blood-retinal barrier within the intra-retinal capillary network. This barrier controls the passage of fluids, ions and molecules and provides protection from pathogens to maintain homeostasis of the neurosensory retinal environment. In diabetic retinopathy (DR), which occurs with an overall prevalence of ~35% in diabetic patients (271), there is a breakdown of the blood-retinal barrier (272). If severe or left untreated, non-proliferative diabetic retinopathy (NPDR) will progress to proliferative diabetic retinopathy (PDR) and/or diabetic macular edema (DME), and these blinding forms of the disease are the leading cause of vision loss in the working-age population (271). Based on the growing prevalence of diabetes mellitus, the number of affected individuals worldwide is expected to rise to >190 million by 2030 (273). Early clinical features of DR include microaneurysms, hemorrhages, venous beading and intra-retinal microvascular abnormalities (IRMAs). PDR is diagnosed upon observation of neovascularization and can co-occur with vitreous and pre-retinal hemorrhage (274–276). DME is classified based on the proximity of mild to severe retinal thickening to the center of the macula. Clinically-significant macular edema (CSME) is determined by OCT imaging (277).

The pathogenic underpinnings of NPDR, PDR and DME are numerous, and substantial progress has been made in the delineation of the cellular, biochemical and molecular mechanisms that drive the disease. Several recent reviews summarize the microvascular, neuroglial, neuronal, immune and outer retinal dysfunctions hypothesized to be key drivers of DR pathogenesis (278–281). Most of our current pathogenic hypotheses are derived from preclinical diabetic models, which are used to examine the histopathological and biochemical changes in retinal tissues due to hyperglycemia and/or hypoxia. Because of these efforts, there are currently several approved treatment options for DR patients including laser photocoagulation, and intravitreal administration of steroids and VEGF inhibitors. Until recently, laser photocoagulation has been the standard of care for DR and DME, slowing progression of vision loss in DR patients (277). A combination of advances in pharmacologic treatment options and observations of significant side effects in patients receiving conventional laser therapy (84) has led to decreased use of these treatments today (282,283).

##### Corticosteroids

Corticosteroids have anti-inflammatory, anti-angiogenic and anti-permeability properties, which is the basis for their use in DR. The exact mechanisms of steroid-induced stabilization of the blood retinal barrier, decreased exudation and decreased inflammatory mediators are unknown. However, as reviewed recently (284), corticosteroid treatment-induced upregulation of lipocortins (eg. phospholipase A2) reduces leukostasis and inhibits the release of inflammatory mediators such as VEGF, TNFα and other chemokines. Currently, there are three approved intravitreally-administered corticosteroids for the treatment of DME; MaQaid® (triamcinolone acetonide, Wakamoto Pharmaceutical), Posurdex®/Ozurdex® (dexamethasone sustained release implant, Allergan plc), and Iluvien® (fluocinolone acetonide intraocular implant, Alimera Sciences Inc.). These agents are potent synthetic glucocorticoids with similar chemical structures and vitreal half-lives between 2 and 3 h in monkey and rabbit models (285). Despite their similarities, each of these corticosteroids activates different genes in different target tissues, which may account for some of the observed clinical differences in safety and efficacy (286,287).

Triamcinolone acetonide was evaluated in several preclinical POC studies prior to its approval for DME in 2012. In 1992, Wilson and colleagues evaluated the effects of sub-tenon or intravitreal triamcinolone acetonide on retinal vascular permeability in female Dutch Belt rabbit eyes subjected to argon-laser retinal photocoagulation to induce blood retinal barrier breakdown. Preventative administration of triamcinolone significantly reduced vitreous leakage (288). Triamcinolone’s efficacy in DME patients, and in additional preclinical models of DR, has been documented and reviewed extensively (289–292). Prior to its approval for use in DME patients, Zhang and colleagues evaluated the efficacy of triamcinolone in male SD diabetic rats aged 10–13 weeks (292). Diabetes was induced by intraperitoneal injection of STZ, a glucosamine-nitrosourea compound that is toxic to insulin-producing beta cells of the pancreas in mammals and induces hyperglycemia when given in a large dose. Approximately one month after induction of diabetes, triamcinolone was administered intravitreally. 48 h post-administration, the authors reported increased retinal thickness (due to preservation of retinal architecture, not edema) and decreased albumin leakage, and these effects were associated with triamcinolone-mediated regulation of pathological expression of VEGF-A and its receptors (292). A study conducted in a rabbit VEGF-induced vasculopathy model found that a single intravitreal administration of 2mg triamcinolone completely blocked retinal and iris leakage for 45 days (291). In this model, recombinant human VEGF_165_ is injected into the vitreous of female Dutch Belt rabbits. Fluorophotometry is used to quantify extent of anterior and posterior vascular leakage, and subjective scoring is used to grade retinal vascular dilation and tortuosity using sodium fluorescein fundus angiography.

Numerous preclinical studies have demonstrated the efficacy of dexamethasone in the reduction of ocular inflammation and retinal vascular leak (reviewed in (284,286)). A VEGF-induced vasculopathy model in NHP is described as a key preclinical POC model in patent documentation for dexamethasone intravitreal implant (293). Cynomolgus monkeys received 700μg dexamethasone or sham treatment via a drug delivery device administered intravitreally. At 1, 7 and 15 weeks post-injection, recombinant human VEGF_165_ was given intravitreally and assessments of anterior chamber flare (slit lamp), retinal vascular leak and dilation (fluorescein fundus angiography), foveal thickness (OCT), optic nerve cup volume (retinal tomography) and electroretinography (ERG) were made 1 week later. The results of this experiment showed that dexamethasone inhibited the development of VEGF-induced retinopathy throughout the 16-weeks experiment.

The effects of fluocinolone acetonide were established in a rabbit model of uveitis prior to the drug’s launch for DME (294). Sustained release devices were delivered to the vitreous cavity of New Zealand White rabbit eyes following a subcutaneous immunization of tuberculin antigen. Uveitis was then induced by intravitreal injection of tuberculin antigen. Anterior chamber flare, cell and vitreous opacity were significantly reduced compared to controls, with faster and more robust inflammation suppression at the higher dose level. Taken together, review of the preclinical models used to establish POC for the use of steroids to treat DME demonstrates high translational value to clinical outcomes, partially owing to a common focus on clinically-relevant inflammation-based endpoints.

##### Anti-VEGFs

The current pharmacologic standard of care for DME and PDR patients is inhibition of VEGF, a key angiogenesis growth factor upregulated during development and under hypoxic conditions. Supportive of a role for VEGF in the pathogenesis of DR is the finding of increased VEGF levels in various ocular compartments in DME and PDR patients (295–298). VEGF and its receptors are expressed in normal vasculature of intraocular tissues, and in DR, chronic hyperglycemia, hypoxia, vasopermeability and thickening of the basement membrane are physiological changes hypothesized to underlie the observed increases in VEGF levels (272,278,299,300). RPE cells synthesize and secrete VEGF, and Muller cell-derived VEGF also plays a role in diabetes-induced inflammation and retinal vascular leakage (301). There are currently two anti-VEGF agents approved for the treatment of DR; Lucentis™ (ranibizumab; Genentech) and Eylea® (aflibercept; Regeneron Pharmaceuticals) (302–304). Avastin® (Bevacizumab, Genentech) is commonly used off-label for DME.

Ranibizumab was first approved by the FDA for the treatment of DME in 2012, and in April 2017, was approved for the treatment of DR without DME. The effects of ranibizumab have been characterized in numerous preclinical models of DR and exudative AMD. Two specific studies are reported in documentation describing preclinical POC prior to its approval for use in DME patients. In hairless guinea pigs, a modified Miles assay showed that ranibizumab co-administered intradermally with recombinant human VEGF_165_ inhibited permeability in a concentration-dependent manner as quantified by leakage of Evans Blue dye into the injection sites (305). A laser-induced CNV model in cynomolgus monkeys was also used to test the efficacy of ranibizumab as described above in the AMD section (219). There are no specific preclinical studies listed in support of ranibizumab’s approval for DR without DME; the rationale for clinical evaluation is provided in findings from clinical studies (306).

Regeneron and licensee Bayer’s aflibercept is approved in the US for treatment of DME (303). A murine oxygen-induced retinopathy (OIR) model demonstrated the efficacy of aflibercept in neonatal mice (307). Aflibercept reduced pathological angiogenesis relative to control mice. A follow-up study in the OIR model showed similar effects of intravitreal injection of aflibercept (308). A canine model of OIR using Beagle dogs was also used to assess the efficacy of aflibercept, and dose-dependent differences in effects on existing neovascularization, revascularization and vascular regression were used as a basis for clinical dose selection (309). The effects of aflibercept on blood retinal barrier breakdown were determined using STZ-induced diabetic SD rats in two reported studies (310,311). Aflibercept administered intravitreally 1 week after induction significantly reduced retinal vascular permeability to levels of non-diabetic rats (310). Similar effects were obtained in the same model when aflibercept was administered subcutaneously, and leukostasis was also significantly reduced (311).

While many patients show slowing of disease progression or vision improvements with anti-VEGF treatment, a substantial number are non- or insufficiently-responsive (302,312,313). Further, patients typically receive repeated intravitreal injections every 1 to 2 months. Like nAMD, two broad strategies aimed at decreasing patient and economic burden focus on either long-acting/sustained delivery of anti-VEGF therapeutics, or exploitation of additional therapeutic targets hypothesized to be disease modifying. For DME, there are two assets currently in clinical development with increased duration of action; brolucizumab (RTH258), a humanized monoclonal single-chain FV antibody fragment targeting VEGF-A, (Alcon Research / Novartis) and abicipar pegol, a PEGylated ankyrin repeat protein (DARPin) targeting VEGF-A (Allergan plc and Molecular Partners).

No specific *in vivo* preclinical POC data are associated with brolucizumab’s development. The *in vivo* preclinical models associated with abicipar pegol’s progression to the clinic include a murine corneal neovascularization model and a rabbit VEGF_165_-induced vasculopathy model (314). The murine corneal neovascularization model is induced by debridement of the corneal epithelium, which causes pathological vessels to grow from the limbus toward to cornea’s center. In the study, mice dosed systemically with abicipar in both prevention and intervention treatment modes showed significantly reduced area of neovascularization. In the rabbit VEGF_165_-induced vasculopathy model, a single intravitreal injection of 125μg abicipar was compared to an equimolar dose of ranibizumab (170μg). A significant reduction of retinal fluorescein leakage was observed in both groups at 2 weeks post-dosing but was only observed in abicipar-treated eyes at 4 weeks. In the same study, two doses of abicipar were evaluated in a rabbit model of chronic retinal neovascularization and leak. The model is induced by intravitreal injection of a gliotoxin, dl-AAA (241,242). In this model, significant leak suppression following intravitreal injection of 230 or 700μg abicipar was observed for up to 10 weeks (314).

##### Drugs Acting on Other Targets

Several therapeutics targeting the Tie2/angiopoietin pathway have recently progressed to clinical development. The Tie2 receptor plays a critical role in vascular stability, as described above. Aerpio’s small molecule inhibitor of VE-PTP, AKB-9778, acts a functional agonist by preventing dephosphorylation of Tie2 and maintaining its agonistic state. Positive effects of VE-PTP inhibition were observed in several murine models; OIR, *Rho*-VEGF-transgenics, modified Miles assay, *Tet-opsin-VEGF* transgenics, and laser-induced CNV. Intraocular or subcutaneous injections of AKB-9778 inhibited ischemia-induced retinal neovascularization in the OIR model, subretinal neovascularization in the *Rho*-VEGF-transgenic mouse model, and CNV in the murine laser-induced CNV model using 6-weeks old C57 BL/6J mice. In the latter, combination treatment with AKB-9778 and aflibercept resulted in greater inhibition of CNV area compared with either treatment alone. Retinal vascular leakage was attenuated in *Tet-opsin-VEGF* transgenic mice treated with AKB-9778, and histamine-, thrombin- or lipopolysaccharide-induced dermal leak (315). Taken together, the results suggest that maintenance of Tie2 agonism stabilizes the vasculature against multiple vessel destabilizing insults and pro-permeability factors.

Regeneron and Roche have both entered the clinic in DME patients with assets targeting Ang2, Tie2’s context-dependent antagonist. Regeneron, in collaboration with Bayer, has developed a co-formulation of their VEGF trap molecule, aflibercept, with an anti-Ang2 antibody, nesvacumab (REGN910–3). Two key preclinical studies provided rationale for progression to the clinic; increased duration of leak inhibition and evidence of neovessel regression and remodeling in the dl-AAA rabbit model using male New Zealand White rabbits (316,317) and decreased total lesion volume in a matrigel CNV rat model using SD rats (318).

Hoffman-La Roche and Chugai have completed a phase 2 trial in DME patients with a bispecific monoclonal antibody developed using CrossMAb technology that inhibits VEGF-A and ANG2. Key preclinical data supporting progression to the clinic were derived from the JR5558 mouse model (319) as well as an NHP laser-induced CNV model (320). The JR5558 spontaneous CNV model is characterized by VEGF-A-driven angiogenesis, focal edema, neural cell loss and dysfunction (321). Neovascularization originates in the choroid between postnatal day 10 and 15 and progresses to the retina by postnatal day 25. Pathological neovascularization, leak and edema are readily observable by color fundus, optical coherence tomography imaging and angiography. The efficacy of anti-VEGF/anti-Ang2 administered systemically starting at postnatal day 45 was compared to equimolar doses of IgG control, anti-VEGF alone and anti-Ang2 alone. *In vivo* imaging at postnatal day 60 and postmortem histology at day 61 showed significant reductions in lesion number and area for all treatment groups, with a greater therapeutic effect observed in anti-VEGF/anti-Ang2-treated animals (319). Similarly, RG-7716 (faricimab) reduced lesion severity significantly more than anti-VEGF alone in the cynomolgus NHP CNV model, which was associated with significant reductions in aqueous humor levels of VEGF, Ang2 and pro-inflammatory cytokines IL-6, IL-8, and MCP-1 (320).

Along with the Tie2/angiopoietin pathway, additional targets are currently being pursued in early clinical development programs for DR. Thrombogenics reported positive results from a phase 2 trial with THR-317, an anti-placental growth factor recombinant humanized neutralizing monoclonal antibody, and preclinical efficacy was established in several animal models, including STZ-induced diabetic C57 BL/6 mice, Akimba mice (5–7 weeks old, male), and a murine laser-induced CNV model induced in 8–10 weeks old male C57 BL/6J mice (322). Stealth BioTherapeutics has completed a phase 1 trial in DME patients treated with elamipretide, a mitochondria-targeting peptide thought to address pathological levels of oxidative stress, and preclinical POC was established in a hydroquinone-induced murine model of mitochondrial dysfunction (323). SciFluor is developing SF-0166, a small molecule inhibitor of alpha-v/beta-3, for DME. Positive topline results were reported in a phase 1/2 trial, and the company published *in vivo* preclinical results in the OIR mouse model and a rabbit model of laser-induced CNV (male Dutch Belt rabbits) (324). Merck/Kalvista is developing KVD-001 (KV123833), a small molecule plasma kallikrein inhibitor for DME. Subcutaneous dosing in a murine model of VEGF-induced vasculopathy was shown to decrease retinal vascular permeability (325). Although there are many different animal models that capture diverse pathological features of DR, the models directly associated with currently-approved drugs and those in active development can be grouped into one of only a few categories, as shown in Table V.

**Table V:** Preclinical Models Used to Establish POC for Diabetic Retinopathy & Diabetic Macular Edema

Model	Species	Pros	Cons	Utility
Miles assay	Guinea pig, C57BL6 mouse	Ability to assess multiple vascular insults Leak from destabilization of mature vasculature Most insults are anti-VEGF responsive	Non-ocular Acute/self-resolving Unrelated vascular bed Difficult to establish anti-VEGF differentiation	Ranibizumab, AKB-9778
Uveitis	New Zealand White rabbit	Large eye facilitates intraocular dosing/PK-PD correlations Leak from destabilization of mature vasculature Anti-VEGF responsive but captures broad inflammatory pathogenic factors	Phenotypic changes not specific to retinal vasculature Edema not commonly reported	Fluocinolone
STZ-induced diabetic rodents, genetic diabetic rodents	Sprague-Dawley, Brown Norway rat, C57BL6 mouse, Akimba mouse	Phenotype caused by hyperglycemia/peripheral metabolic-like syndrome Anti-VEGF-responsive Amenable to evaluating anti-inflammatory therapies	Variable leak & edema phenotype Lengthy induction time Difficult intraocular dosing Difficult to establish anti-VEGF differentiation	Triamcinolone, Aflibercept, THR-317, Lifitegrast
VEGF-induced vasculopathy	Dutch Belt rabbit, Cynomolgus monkey, C57BL6 mouse, (*Rho*-VEGF transgenics, *Tet-opsin*-VEGF transgenics)	Large eye versions facilitate intraocular dosing/PK-PD correlations Leak from destabilization of mature vasculature quantifiable *in vivo* Anti-VEGF responsive	Acute/self-resolving (direct VEGF injections) Edema not commonly reported VEGF-driven does not capture additional pathogenic factors Small eye versions limit ability to test intraocular dosing Difficult to establish anti-VEGF differentiation	Triamcinolone, Dexamethasone, Abicipar, AKB-9778, KV123833
Laser-induced CNV	Cynomolgus monkey, Dutch Belt rabbit, C57BL6 mouse	Large eye versions facilitate intraocular dosing/PK-PD correlations Leak quantifiable *in vivo* & ex vivo Anti-VEGF responsive	Self-resolving injury model Choroidal phenotype Edema not commonly reported Proliferative phenotype Difficult to establish anti-VEGF differentiation	Triamcinolone, Ranibizumab, RG-7716 (faricimab), SF-0166, THR-317
Spontaneous CNV	JR5558 mouse	Edema reported in some studies Leak quantifiable *in vivo* & ex vivo Anti-VEGF responsive	Difficult intraocular dosing Proliferative phenotype Difficult to establish anti-VEGF differentiation	RG-7716 (faricimab)
dl-AAA-induced persistent retinal vascular leak	Dutch Belt, New Zealand White rabbit	Chronic model enabling duration of action studies Large eye facilitates intraocular dosing /PK-PD correlations	Proliferative phenotype Edema not observed	Abicipar, Nesvacumab
		Retinal vessels affected although differ substantially *vs*. humans Leak quantifiable *in vivo* Anti-VEGF responsive	Difficult to establish anti-VEGF differentiation	
Corneal neovascularization	C57BL6 mouse	Vascularization readily observable Anti-VEGF responsive	Self-resolving injury model Unrelated vascular bed affected Systemic dosing required Difficult to establish anti-VEGF differentiation	Abicipar
Oxygen-induced retinopathy	C57BL6 mouse, Beagle dog	Anti-VEGF responsive Recapitulates pathological neovascularization in response to hypoxia insult	Difficult intraocular dosing Proliferative phenotype Edema not commonly-reported Difficult to establish anti-VEGF differentiation	Aflibercept, AKB-9778, SF-0166

## Considerations For Preclinical Models Of Retinal Diseases

There are key factors that must be considered in the choice of the model as well as the predictive utility of the effect observed in a given preclinical model of retinal disease. These include complexity of pathogenesis/ target mechanism of action, species specificity for testing biologics, structural differences between preclinical species and humans, duration of disease etiology/ progression and, critically, translational validity of endpoints.

The first consideration starts with the experimental drug’s target and hypothesized mechanism of action. Although extremely useful for evaluating therapeutics which target the VEGF pathway, it is difficult to demonstrate additive effects of non-VEGF anti-angiogenic strategies using VEGF-driven models or models that are highly-responsive to inhibition of VEGF. *In vivo* models of retinal vascular dysfunction and hyperpermeability that show anti-VEGF resistance or lack of phenotype attenuation with anti-VEGF treatment are greatly needed. Similarly, no preclinical models have been validated by positive phase 3 clinical data, likely owing to the complex and elusive etiology of GA and glaucomatous optic neuropathy. There is a need for preclinical models that enable drug developers to test potential compounds against multiple targets for these multifactorial diseases.

Additional consideration must be given to the types of pathological features captured by the *in vivo* model, and whether the preclinical endpoints translate to commonly-used clinical endpoints. While spontaneous, laser-, matrigel- or gliotoxin-induced models of pathological neovascularization may recapitulate some of the features of PDR and nAMD, it’s unlikely that the preclinical endpoints used, such as area of neovascularization or extent of vascular regression, are useful predictors of clinical outcomes in NPDR and DME in which visual acuity, Diabetic Retinopathy severity scale (DRSS) score, and central subfield thickness are used to evaluate efficacy. Further, when vascular permeability measures are used in proliferative models, they are capturing exudation from newly-formed pathological vessels *versus* exudation caused by destabilization of mature vasculature, such as that observed in DME. It is of note that a primary endpoint used widely in clinical trials is change in visual acuity from baseline, for which there does not exist a similar, translatable endpoint in current preclinical paradigms. The increased use of optical coherence tomography-angiography (OCT-A) imaging in clinical and experimental settings will likely improve our ability to detect subtle microvascular changes, like capillary drop-out and areas of non-perfusion, both in patients and in animal models. Consensus around a DRSS-like scoring system for color fundus and fundus angiography images in rodents, rabbits and nonhuman primates that maps, as best possible, onto the clinical DRSS scoring system, could be helpful in comparing relative efficacy of novel therapeutics across labs and in serendipitous identification of mechanisms of action.

Species cross-reactivity is an important factor in considering translation of preclinical results to the clinic, especially given the recent increase in development of biologics for retinal disease. Humanized antibodies may have little or no efficacy in preclinical models due to insufficient cross-reactivity. Binding affinity for the specific target must be verified in the species in which preclinical efficacy testing will be conducted. If cross-reactivity is not confirmed, surrogate molecules will be necessary to validate targets in animal models. For example, surrogate molecules were used in Roche’s preclinical evaluation of RG7716 the murine spontaneous CNV model (246).

Another critical challenge that stems from the practical time and cost considerations of animal studies is the ability to model chronic diseases in the laboratory setting. While the CNV model has been the most widely used preclinical model for nAMD, being an acute injury model, it is not representative of human pathogenesis and the disease, which occurs over a more chronic period. STZ-induced and genetic models of Type 1 or Type 2 diabetes may mimic some of the effects of chronic hyperglycemia in the retina, albeit on a much shorter timescale. To date, pharmacological interventions for GA and glaucomatous neuroprotective strategies with established preclinical POC have failed to translate to efficacy in clinical trials. Most preclinical models of GA show rapid retinal degeneration and therefore fail to recapitulate the progressive nature of the disease. Use of progressive models like the blue light irradiation model in NHP used to establish preclinical POC for brimonidine may be more suitable because progressive cyto- and neuro-degeneration more likely to capture mechanisms driving GA in humans. In addition, endpoints analogous to those used in the clinic, such as growth of lesion from baseline, can be employed. A similar challenge has been observed with neuroprotective strategies in optic neuropathy as most of the current models induce degeneration via an acute trauma which may not accurately recapitulate all features of a slowly progressive optic neuropathy. Clinical success in complex neurodegenerative diseases needs implementation of novel surrogate endpoints which can be accurately translated from animals to humans.

Perhaps because of this shortened timescale and/or anatomical differences between rodent and human eyes, the severity of retinopathy in preclinical models is variable and inevitably not as severe as that seen in patients with vision-impairing DR. For example, retinal edema is not a robust feature of any of the most commonly-used preclinical DR models despite this being a primary clinical endpoint in many past and ongoing DME trials. Recently, foveal edema in aged, spontaneously-diabetic NHPs was described (326). While this is an exciting prospect given the similarities in disease pathogenesis and anatomy to human patients, both ethical and practical considerations will limit this model’s widespread use. Given the focus on sustained release and extended duration anti-VEGF assets due to the burden in AMD and DR patients caused by frequent IVT injections and trips to the clinic, large eye models of long-term vascular instability and exudation, such as the dl-AAA rabbit model, will be helpful in predicting clinical duration of action. Additionally, there is a need for better techniques to follow disease progression and effects of therapeutic agents in preclinical models for both nAMD and GA over long periods of time. Newer outcome measures like the area of drusen growth have been established (eg. eculizumab and lampalizumab trials) and would be useful to add as preclinical endpoints allowing for comparisons of animal and human efficacy.

## CONCLUSIONS

Here we have provided a summary of key POC preclinical models used in the development of therapies approved or under evaluation for common ocular diseases. Multiple preclinical models are needed to capture different aspects of multifactorial diseases of the eye. While one model may be appropriate to define pathogenic mechanisms of the disease and identify new drug targets, another model might be needed to evaluate a novel drug’s duration of action, pharmacokinetic/pharmacodynamic relationship, and its effect on clinically-relevant endpoints. Another common challenge in the laboratory setting is the ability to mimic chronic disease states in a tractable testing modality. Existing preclinical models have been critical in the development of vision-sparing symptomatic treatments; however, their utility may be limited in the context of development of disease-modifying drugs. Furthermore, preclinical demonstration of efficacy of a novel drug in preventative treatment mode might not translate in clinical populations already undergoing disease progression, in which the new drug must intervene to slow or halt its progression. Successful development of next generation therapies in ocular diseases will need improved models that capture key clinical features and can be assessed with translational endpoints. Applying clinical biomarkers and endpoints to preclinical models will aid in bridging the gap between these two domains.

## ABBREVIATIONS

AAA: Alpha-aminoadipic acid
AMD: Age-related macular degeneration
Ang1: Angiopoietin-1
Ang2: Angiopoietin-2
AqH: Aqueous humor
BCVA: Best corrected visual acuity
C5: Complement factor 5
CFD: Complement factor D
CNV: Choroidal neovascularization
CSME: Clinically-significant macular edema
DED: Dry eye disease
DME: Diabetic macular edema
DR: Diabetic retinopathy
DRSS: Diabetic Retinopathy Severity Scale
ECM: Extracellular matrix
ERG: Electroretinography
GA: Geographic Atrophy
IOP: Intraocular pressure
IRMAs: Intra-retinal microvascular abnormalities
IVT: Intravitreal
KCS: Keratoconjunctivitis sicca
MGD: Meibomian gland dysfunction
nAMD: Neovascular age-related macular degeneration
NDA: New drug application
NHP: Non-human primates
NPDR: Non-proliferative diabetic retinopathy
OCT: Optical coherence tomography
OIR: Oxygen-induced retinopathy
ONL: Outer nuclear layer
PACG: Primary angle-closure
PDGF: Platelet derived growth factor
PDR: Proliferative diabetic retinopathy
POAG: Primary open angle
POC: Proof of concept
RGCs: Retinal ganglion cells
RPE: Retinal pigment epithelium
SD: Sprague Dawley
STZ: Streptozotocin
VEGF: Vascular endothelial growth factor
VE-PTP: Vascular endothelial-protein tyrosine phosphatase
